# Alternative splicing of *Drosophila Nmnat* functions as a switch to enhance neuroprotection under stress

**DOI:** 10.1038/ncomms10057

**Published:** 2015-11-30

**Authors:** Kai Ruan, Yi Zhu, Chong Li, Jennifer M. Brazill, R. Grace Zhai

**Affiliations:** 1 Department of Molecular and Cellular Pharmacology, University of Miami Miller School of Medicine, Miami, Florida 33136, USA; 2 Program in Molecular and Cellular Pharmacology, University of Miami Miller School of Medicine, Miami, Florida 33136, USA; 3 Program in Human Genetics and Genomics, University of Miami Miller School of Medicine, Miami, Florida 33136, USA

## Abstract

Nicotinamide mononucleotide adenylyltransferase (NMNAT) is a conserved enzyme in the NAD synthetic pathway. It has also been identified as an effective and versatile neuroprotective factor. However, it remains unclear how healthy neurons regulate the dual functions of NMNAT and achieve self-protection under stress. Here we show that *Drosophila Nmnat* (*DmNmnat*) is alternatively spliced into two mRNA variants, RA and RB, which translate to protein isoforms with divergent neuroprotective capacities against spinocerebellar ataxia 1-induced neurodegeneration. Isoform PA/PC translated from RA is nuclear-localized with minimal neuroprotective ability, and isoform PB/PD translated from RB is cytoplasmic and has robust neuroprotective capacity. Under stress, RB is preferably spliced in neurons to produce the neuroprotective PB/PD isoforms. Our results indicate that alternative splicing functions as a switch that regulates the expression of functionally distinct *DmNmnat* variants. Neurons respond to stress by driving the splicing switch to produce the neuroprotective variant and therefore achieve self-protection.

## Abstract (web summary)

Nicotinamide mononucleotide adenylyltransferase (NMNAT) acts in the NAD biosynthesis pathway and has neuroprotective activity. Ruan *et al*. show that the neuroprotective activity of NMNAT is restricted to a splice variant of the enzyme, and that this variant is preferentially spliced in response to stress.

## Graphical abstract

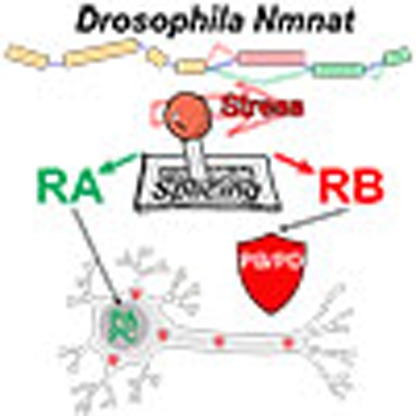

Nicotinamide mononucleotide adenylyltransferase (NMNAT) is a highly conserved housekeeping enzyme catalysing reversibly the last step in the NAD salvage pathway in all living organisms^1^. In addition, NMNAT proteins are essential for neuronal maintenance and protection^2^. Functional studies in *Drosophila* and mammals have shown that loss of NMNAT causes neurodegeneration. For example, loss of *DmNmnat* in photoreceptors causes retinal degeneration^2^; loss-of-function mutations in human *Nmnat1* cause Leber congenital amaurosis 9 (LCA9)^3^,^4^,^5^,^6^, a childhood blindness disease that involves degeneration of the photoreceptors^3^,^5^,^7^; and loss of mouse *Nmnat2* causes axonal degeneration^8^. The neuronal maintenance function of NMNAT can be enhanced by overexpression to achieve neuroprotection against a variety of neurodegenerative conditions^9^. For example, overexpression of yeast NMNAT (NMA1) protects against polyglutamine (polyQ)-induced cytotoxicity^10^, expression of *Drosophila* NMNAT protects against light-induced retinal degeneration^2^ or Wallerian degeneration in cultured rat dorsal root ganglion neurons^11^ and ectopic expression of any of the three human NMNAT confers neuroprotection against axonal degeneration in cultured neurons or transgenic mouse models (see reviews^9^,^12^). These observations suggest that the neuroprotective property of NMNAT is conserved through distant phyla. In most of the mammalian degenerative models, particularly in axonal degeneration, it has been suggested that the NAD synthetic activity of NMNAT is required for its neuroprotective activity (see reviews^12^,^13^). In addition to its enzymatic function, work from our group^14^ and others^10^,^15^ has identified a chaperone function of *Dm*NMNAT that contributes to neuroprotection by directly interacting with unfolded/misfolded proteins in the nervous system, reducing the load of toxic protein oligomers and facilitating the clearance of unfolded proteins^14^,^16^,^17^. These studies in different model systems have provided evidence supporting two roles of NMNAT in neurons: chaperone and NAD metabolism. It remains unclear whether, and if so how, neurons regulate the partitioning of NMNAT into these two roles and balance metabolic and self-protective activities.

Alternative splicing is an important post-transcriptional regulatory mechanism for cells to generate transcript variability and proteome diversity^18^,^19^. It is estimated that 95% of multiexonic genes in humans and 65% in *Drosophila* are alternatively spliced^20^,^21^. In the nervous system, this process is especially common for gene regulation. Recent work has identified several aspects of neural development and function that are specifically controlled by alternative splicing. Examples include fibroblast growth factor 8 in neural tube patterning^22^, the *Drosophila* homologue of Down's syndrome cell-adhesion molecule in the axon guidance and dendrite morphogenesis^23^,^24^,^25^, as well as neurexins and neuroligins in synaptogenesis^26^,^27^. In the mature nervous system, it has been shown that Nova-1 regulates neuronal-specific alternative splicing and is essential to maintain neuronal health^28^, and neuronal activity dynamically regulates the splicing of products that determine neuronal excitation such as SNARE protein SNAP25 (ref. 29). Misregulation of alternative splicing or of splicing regulatory factors can lead to multiple human neurodegenerative diseases^30^, such as spinal muscular atrophy^31^, frontotemporal lobar dementias and amyotrophic lateral sclerosis^32^, and tauopathies^33^. These studies highlight the important role of alternative splicing in nervous system development and function. However, the role of alternative splicing in neuronal maintenance and protection has not been as widely studied.

Here we seek to address the mechanisms of how neurons regulate the expression of *DmNmnat* to enhance its neuroprotective capability. We have characterized the neuroprotective functions and biochemical and cellular properties of *Dm*NMNAT isoforms, and directly monitored the shift in alternative splicing *in vivo* in the *Drosophila* nervous system using an alternative-splicing reporter. Our results identify alternative splicing of *DmNmnat* as a switch for regulating neuroprotection, and reveal a neuronal mechanism of achieving NMNAT-mediated self-protection under stress.

## Results

### *DmNmnat* is alternatively spliced

**Figure 1:**
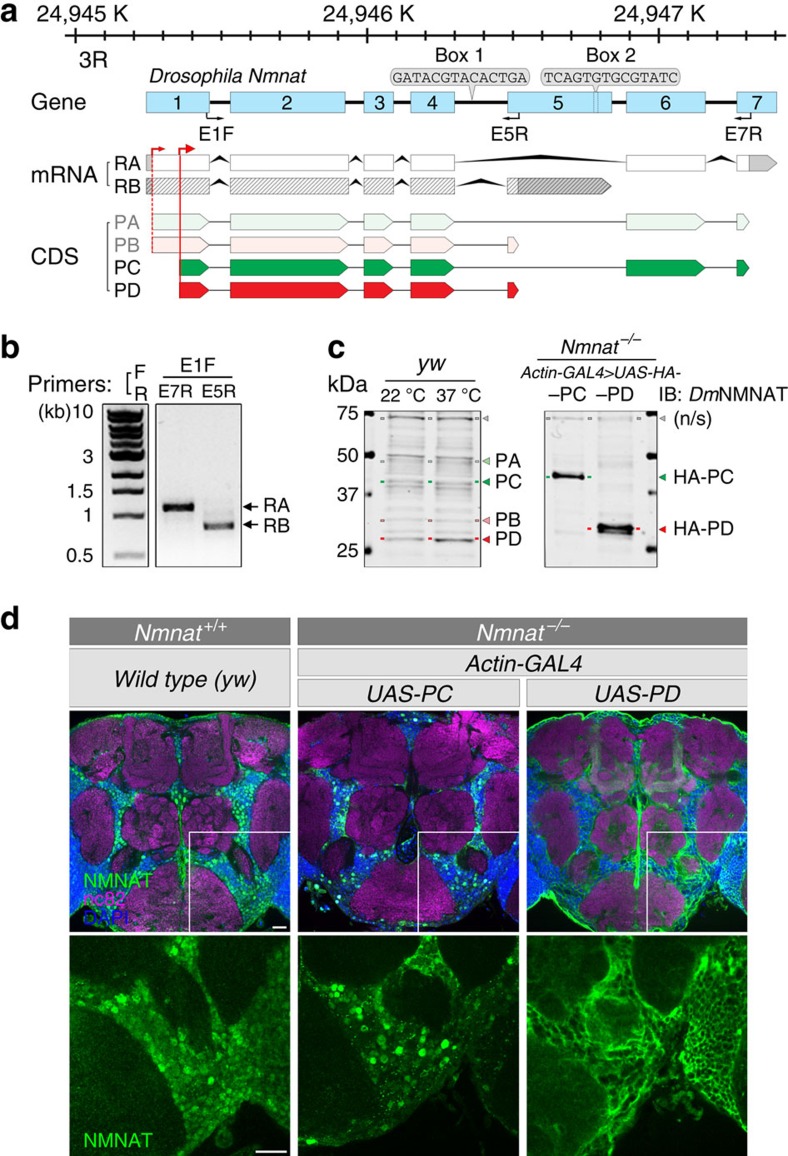
*DmNmnat* is alternatively spliced into two major mRNA variants and possibly four protein isoforms. (**a**) Diagram of gene structure of *DmNmnat* and predicted expression products. Boxes 1 and 2 mark the sequences that form the stem–loop structure that is required for alternative splicing^34^. Black arrows indicate the sequence locations of forward (E1F) and reverse (E5R, E7R) primers used. Red arrows mark the translation start sites. (**b**) RT–PCR of total RNA from head extracts of wild-type flies (*yw*) using two primer sets as indicated in **a**. (**c**) Western blot analysis using a polyclonal *Dm*NMNAT antibody^2^ of head extracts from wild-type flies (*yw*) under 22 and 37 °C, or flies overexpressing PC and PD isoforms with ubiquitous *actin-GAL4* driver in *Nmnat*^*−/−*^ null background. Grey arrowheads indicate likely nonspecific bands. (**d**) Adult fly brains from wild-type (*yw*) or *Nmnat*^*−/−*^ null flies expressing PC or PD were probed for NMNAT (green), synapse marker nc82 (magenta) and DAPI (blue). Bottom row shows the respective NMNAT channel of the boxed area in top row. Scale bars, 20 μm for each row.

*Drosophila melanogaster* has one *Nmnat* gene that contains seven tandem exons (Fig. 1a). Gene structure analyses suggest that *DmNmnat* is alternatively spliced into two mRNA variants RA and RB, with the first four exons constitutively included and the last three exons differentially spliced (Fig. 1a). A previous report analysing splice sites in the *Drosophila* genome identified sequences in the fourth intron and fifth exon that can form a ‘stem–loop' structure to facilitate the alternative splicing event (Boxes 1 and 2 in Fig.1a)^34^. In addition, the first exon contains two putative translational start codons, which may further increase the protein diversity. To determine the endogenous *DmNmnat* variants, we first used variant-specific amplification primers located in exon 5 (E5R) and 7 (E7R) to identify the mRNA transcripts. Two mRNA transcripts from adult fly brain extracts were identified (Fig. 1b) and confirmed by Sanger sequencing to correspond to RA and RB mRNA variants, as annotated in FlyBase (www.flybase.org).

Two protein products are predicted to translate from two alternative start codons for each mRNA variant: PA/PC from RA and PB/PD from RB (Fig. 1a). To identify the protein isoforms, we performed western blot analysis using a *Dm*NMNAT polyclonal antibody^2^. Since the antibody was raised against the PD isoform^2^, we first tested whether this antibody can recognize all four potential protein isoforms. First, we generated and expressed DsRed-tagged *Dm*NMNAT isoforms in HEK293T cells and found that both *Dm*NMNAT and DsRed antibodies recognized all four isoforms (Supplementary Fig. 1a). Next, we generated transgenic fly strains carrying the cDNA of a predicted protein isoform: PA, PB, PC or PD. When the individual transgenes were ubiquitously expressed (*actin-GAL4*) in wild-type (*Nmnat*^*+/+*^) or null (*Nmnat*^*−/−*^) background, four protein isoforms PA, PB, PC and PD were detected at their respective predicted molecular weights: PA (43.8 kD) and PB (33.5 kD) translated from the first start codon, and PC (40.1 kD) and PD (29.8 kD) translated from the second start codon (Supplementary Fig. 1b). The polyclonal NMNAT antibody detected the endogenous NMNAT isoforms and found the expression to increase on heat shock, especially the PB and PD isoforms (Fig. 1c and Supplementary Fig. 1b), consistent with a previous study^35^. Notably, when the transgenes carrying PA or PB cDNA were expressed, PC or PD proteins were also detected at a relatively higher level (Supplementary Fig. 1b), suggesting that the translation from the second translational start codon is more efficient. Sequence analysis of the N-terminal region revealed that the second start site contains a frontal *Drosophila* Kozak consensus sequence (GAAA)^36^, consistent with more efficient translation. To further confirm the functionality of each isoform, we carried out rescue analysis by expressing each isoform ubiquitously in the null background^2^. Expression of individual isoforms suppressed the lethality caused by loss of *DmNmnat*, indicating that all isoforms are functional when expressed alone (Supplementary Table 1). The difference in rescue efficiency was likely owing to differences in transgene expression levels (Supplementary Fig. 1b). Because PC and PD are the dominant isoforms *in vivo*, for the following analyses we focused on the PC and PD isoforms as the model protein products from alternatively spliced mRNA variants, RA and RB, respectively.

### *Dm*NMNAT isoforms have distinct subcellular localizations

Our previous study found endogenous *Dm*NMNAT to be ubiquitously distributed in the cytoplasm and synaptic terminals with enrichment in the nucleus^2^. As these localization observations were made using the polyclonal antibody that recognizes all isoforms (Fig. 1c and Supplementary Fig. 1b), it is possible that such a broad localization pattern is actually the summation of distinct subcellular distributions of each isoform. To address the localization patterns *in vivo*, we ubiquitously expressed PC or PD isoform in *Nmnat*^*−/−*^ background to avoid the influence of endogenous *Dm*NMNAT. PC- or PD-singly expressed adult brains showed a nonoverlapping pattern, where PC is highly enriched in the cell body and PD is predominantly cytoplasmic (Fig. 1d and Supplementary Fig. 2). Therefore, the endogenous *Dm*NMNAT expression pattern is a combined distribution patterns of all isoforms. These data suggest that alternative splicing of *DmNmnat* produces two protein products that localize to distinct subcellular compartments: nuclear PC and cytosolic PD.

**Figure 2:**
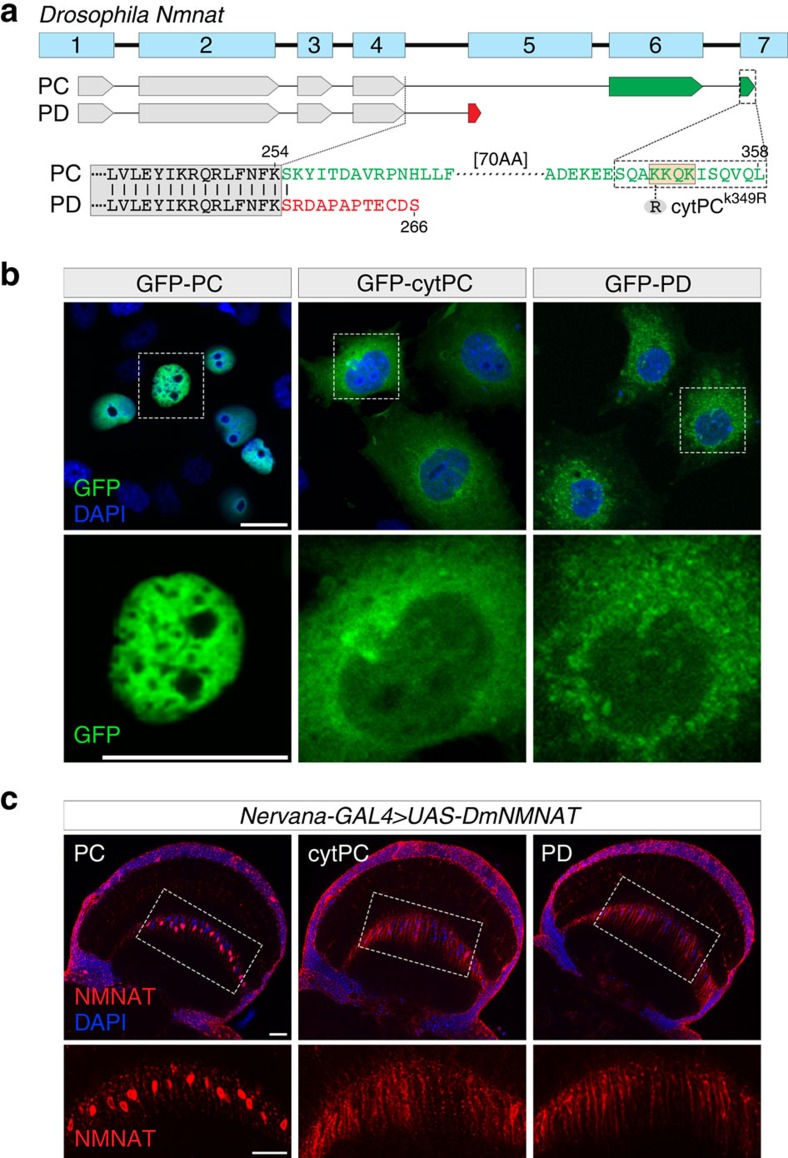
Compartmental localization of PC and PD isoforms in cultured cells and in fly brains. (**a**) Protein sequence alignment between PC and PD isoforms. The nuclear retention element is indicated by box (orange). cytPC^K349R^ is a single point mutation from lysine to arginine. (**b**) Cos-7 cells were singly transfected with GFP-tagged cDNA of PC, cytPC or PD and imaged at 48 h after transfection. DAPI (blue) staining was used to mark the nucleus. (**c**) Adult fly brains expressing PC, cytPC or PD with *nervana-GAL4* were probed for NMNAT (red) and DAPI (blue). Bottom row shows the respective NMNAT channel of the boxed area in top row. Scale bars, 20 μm for each row.

To further investigate the mechanism of *Dm*NMNAT localization, we analysed the C-terminal amino-acid sequence of PC and PD and identified a putative nuclear localization signal, a KKQK motif^37^,^38^ encoded in exon 7 (Fig. 2a). We subsequently generated a mutant PC isoform with the first lysine in the motif mutated to arginine (PC^K349R^) and expressed green fluorescent protein (GFP)-tagged *Dm*NMNAT isoforms in Cos-7 cells. As shown in Fig. 2b, PC was nuclear-localized, while PC^K349R^ was localized to the cytoplasm, suggesting that the KKQK motif is the nuclear localization signal. We named the PC^K349R^ mutant isoform cytPC and generated a transgenic fly strain carrying UAS-cytPC for *in vivo* analysis. Next, we examined the localization of PC, cytPC and PD isoforms *in vivo*. As shown in Fig. 2c, when expressed with a pan-neuronal driver *nervana-GAL4*, PC is highly enriched in the cell body, while cytPC and PD are predominantly cytoplasmic, consistent with the localization pattern in transfected cells. Collectively, these data indicate that *Dm*NMNAT isoforms are localized to distinct cellular compartments because of the differentially spliced C-terminal sequences that result in either the inclusion (PC) or exclusion (PD) of the KKQK nuclear localization motif.

### *Dm*NMNAT isoforms have divergent neuroprotective effects

**Figure 3:**
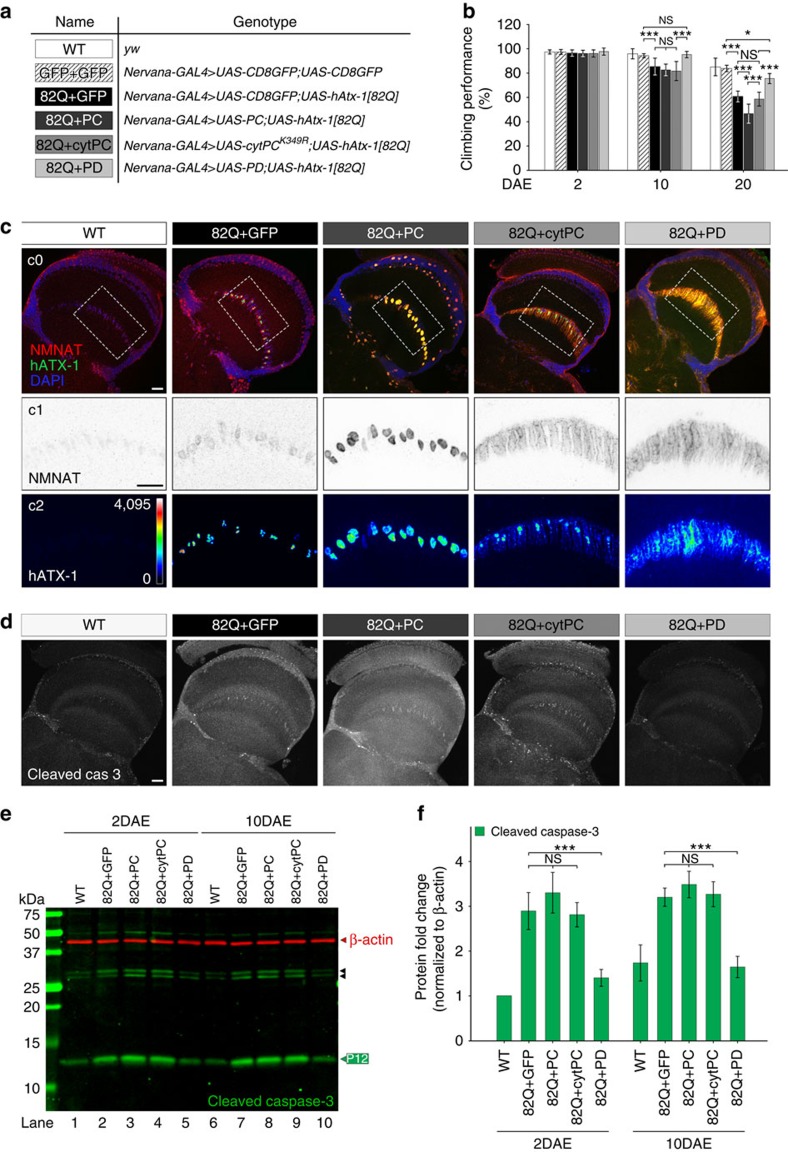
*Dm*NMNAT isoforms have divergent neuroprotective capacities in a *Drosophila* model for SCA1. (**a**) List of genotypes of the transgenic flies examined. Human ataxin-1 with an expanded (82) polyQ tract (*UAS-hAtx-1[82Q]*), and GFP (*UAS-CD8GFP*) or *Dm*NMNAT isoforms PC (*UAS-PC*), cytPC (*UAS-cytPC*^*K349R*^) or PD (*UAS-PD*) were expressed by the pan-neuronal driver *nervana-GAL4.* (**b**) Climbing performance of wild-type, GFP overexpressing flies and hAtx-1[82Q]-expressing flies that co-expressed GFP, PC, cytPC or PD at the ages of 2, 10 and 20 DAE. Ten groups (10 flies in each, total 100 flies) of each genotype and age were tested. All data were presented as mean±s.d. *n*=10. Significance level was established by one-way analysis of variance (ANOVA) *post hoc* Tukey's test. *P≤0.05, ***P≤0.001. (**c**) Adult (2 DAE) female brains of wild-type, or hAtx-1[82Q]-overexpressing flies, were probed for hAtx-1 (green), NMNAT (red) and DAPI (blue). Top row (c0) shows the merged three channels of the *Drosophila* optic lobes. Middle (c1) and bottom (c2) rows are the single channel view of the boxed area in the top row showing the expression and localization of *Dm*NMNAT (c1) and hAtx-1 (c2, intensity indicated with heat maps). Scale bars, 20 μm for each row. (**d**) Adult (2 DAE) female brains of wild-type or hAtx-1[82Q]-overexpressing flies were probed for cleaved caspase-3. Scale bar, 20 μm. (**e**,**f**) Western blot analysis (**e**) and quantification (**f**) of proteins extracted from 2 DAE or 10 DAE wild-type or hAtx-1[82Q]-overexpressing fly heads. For quantification, P12 was considered as cleaved caspase-3 (green box): fold change relative to each level in 2 DAE wild-type flies. β-Actin (red) was used as an internal control. Black arrowheads indicate the intermediate cleavage fragments. All data were presented as mean±s.d., *n*=3. Significance level was established by one-way ANOVA *post hoc* Bonferroni test. ***P≤0.001.

Work from our group and others has shown the robust neuroprotective activity of *Dm*NMNAT, specifically of the PD isoform^14^,^15^,^16^,^39^,^40^. Detecting the endogenous expression of *Dm*NMNAT isoforms compelled us to ask whether all isoforms share similar neuroprotective activity. We tested the neuroprotective efficacy of each isoform against neurodegeneration induced by the expression of human Ataxin-1 with 82 polyQ expansion (hAtx-1[82Q])^14^. Consistent with a previous report^14^, hAtx-1[82Q] expression in the nervous system induced an age-dependent decline in climbing performance, which was partially suppressed by PD expression (82Q+PD). Unexpectedly, PC expression (82Q+PC) not only failed to suppress the behavioural decline, but rather aggravated it (Fig. 3a,b). Interestingly, flies expressing cytPC (82Q+cytPC) showed climbing behaviour similar to the control GFP-expressing flies (82Q+GFP).

To examine the cellular changes during neurodegeneration, we analysed the brain morphology of flies 2 DAE (days after eclosion). This age was chosen because it preceded the onset of behavioural defects (Fig. 3b) and therefore would allow the identification of early events and causal cellular processes of degeneration. Interestingly, the distribution of hAtx-1[82Q] protein differed greatly among the four genotypes tested. Human ataxin-1[82Q] is nuclear-localized and is known to cause nuclear aggregation^41^. As shown in Fig. 3c, in GFP control brains we observed hAtx-1[82Q] in the nuclei surrounded by endogenous NMNAT. In PC-expressing brains, nuclear hAtx-1[82Q] aggregates became larger and co-localized with NMNAT. In cytPC-expressing brains, hAtx-1[82Q] showed enrichment in the nuclei similar to GFP control brains. In contrast, in PD-expressing brains, hAtx-1[82Q] was diffused throughout the neuronal cytoplasm with minimal aggregation in the nucleus.

To determine whether expression of *Dm*NMNAT isoforms affects neuronal cell death by hAtx-1[82Q] expression, we measured the activation of caspase-3 as a hallmark of apoptosis^42^. As shown in Fig. 3d, the level of activated cleaved caspase-3 signal is highest in PC-expressing brains and lowest in PD-expressing brains. To quantitatively monitor the activation of apoptosis, we next examined the biochemical processing of caspase-3. The activation of procaspase-3 involves multiple cleavage steps, resulting in different processed products, and the fully processed product P12 is the activated caspase-3 (ref. 43). As shown in Fig. 3e,f, hAtx-1[82Q] expression induced a significant elevation of P12 levels in brains, indicating hAtx-1[82Q]-induced apoptosis. Specifically, PD expression greatly reduced the activation of caspase-3 to a similar level in the wild-type brains of the same age. However, neither PC nor cytPC expression reduced cleaved caspase-3 (Fig. 3e,f).

Collectively, these results suggest that PD has robust neuroprotective activity, while PC has minimal neuroprotective activity and may even exacerbate hAtx-1[82Q]-induced degeneration as shown by the significant behavioural decline at 20 DAE. Furthermore, a lack of protection by cytPC indicates that the difference in protection between PC and PD is inherent to protein properties beyond subcellular localization.

**Figure 4:**
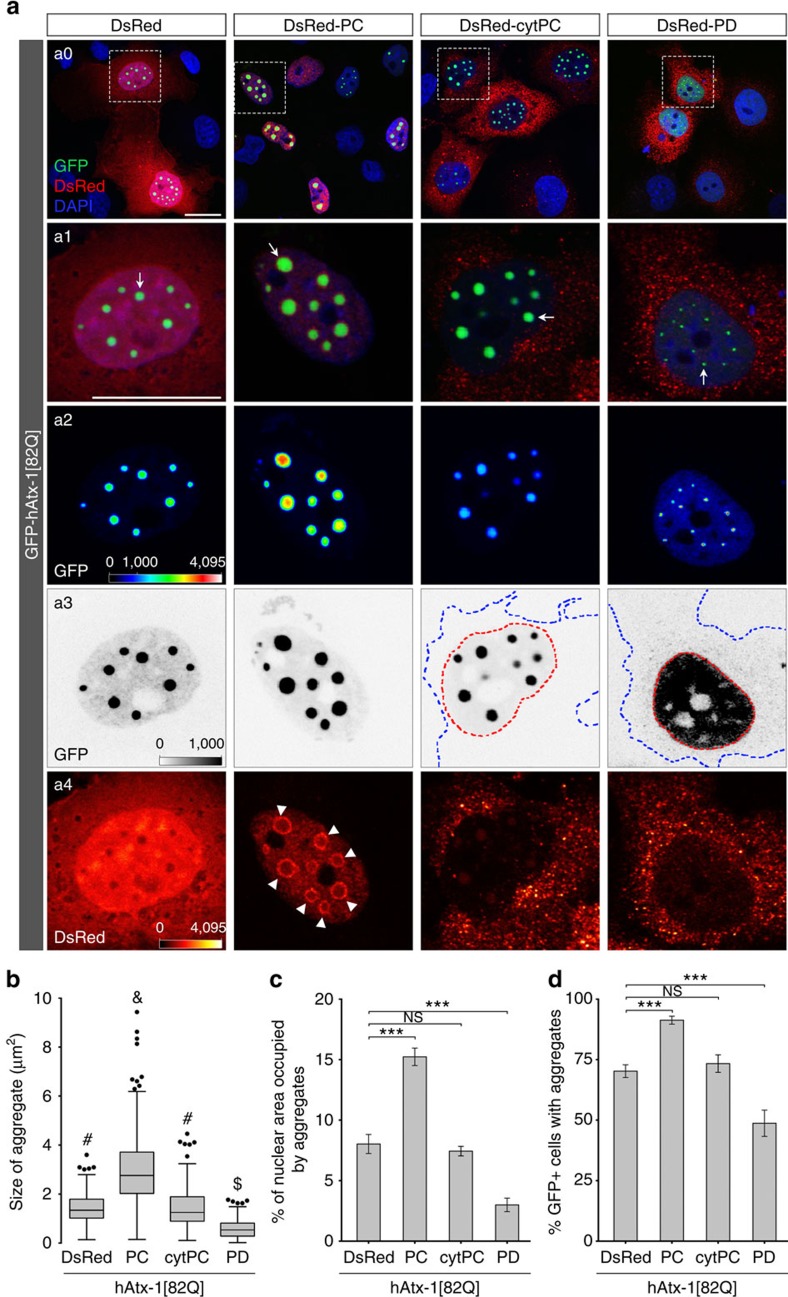
PC promotes the nuclear aggregation of human ataxin-1 with polyQ expansion. (**a**) Cos-7 cells were co-transfected with GFP-hAtx-1[82Q] and DsRed, DsRed-PC, DsRed-cytPC or DsRed-PD, and imaged at 48 h after transfection. DAPI (blue) stain was used to mark the nucleus. (**a1**) Row shows the merged channels of the boxed area in a0 row. a2,a3 rows are the GFP channel view of the boxed area in the a0 row showing the expression and localization of hAtx-1 (a2, intensity indicated with heat maps; a3, overexposed images under inverted grey scale, red and blue dashed lines indicate the boundaries between nucleus and cytoplasm). Bottom row a4 shows the DsRed channels of the boxed area in the a0 row, intensity indicated with heat maps. Scale bars, 20 μm for each row. (**b**–**d**) Quantifications of aggregate size (**b**), percentage of nuclear area occupied by aggregates (**c**) and percentage of GFP-positive cells containing aggregates (**d**). The sizes of 200 aggregates in **b** were plotted with box and whisker plots. Box plots indicate the 25th percentile (bottom boundary), median (middle line), 75th percentile (top boundary) and nearest observations within 1.5 times of the interquartile range (whiskers). Different superscripts are statistically significant at *P*≤0.05, one-way ANOVA *post hoc* Bonferroni test. Nuclear area in **c** is defined by the DAPI channel with a threshold of 400–4,095. Aggregates are defined as objects at least 0.4 μm^2^ and 3,000 intensity units. All data in **c**,**d** were presented as mean±s.e.m., *n*≥20. Significance level was established by one-way ANOVA *post hoc* Bonferroni test. ***P≤0.001.

It has been shown that the size of polyQ aggregates is positively correlated with disease severity and onset^41^,^44^. Previous studies have shown that transgenic hAtx-1[82Q] forms aggregates in cultured cells, transgenic mouse and fly brains and the observed hAtx-1[82Q] puncta clusters correlate with polyQ aggregates^14^,^45^,^46^. To further quantitatively evaluate the divergent effects of *Dm*NMNAT isoforms in nuclear polyQ aggregation, we expressed GFP-tagged human ataxin-1 without polyQ expansion (hAtx-1[2Q]), or with subpathological (hAtx-1[30Q]) or pathological (hAtx-1[82Q]) lengths of polyQ expansion with DsRed-tagged *Dm*NMNAT isoforms in cultured Cos-7 cells. We found that all hAtx-1 proteins formed aggregates (Fig. 4a and Supplementary Fig. 3a), consistent with a previous report^14^. The size of the visible aggregates (arrow), as well as the percentage of cells that contain aggregates, increased with the expansion of the polyQ^47^. Interestingly, co-expression of PC or PD had opposite effects on polyQ aggregation—PC promoted the size and the formation of aggregates in cells, while PD reduced both parameters (Fig. 4 and Supplementary Fig. 3). Notably, PC formed a ‘ring-like' pattern surrounding the nuclear hAtx-1[82Q] aggregates (arrow heads, Fig. 4a4). PD not only reduced the size of nuclear polyQ aggregates but also promoted the partial relocation of hAtx-1 into the cytoplasm, which can be detected when the low intensity range (0–1,000) was amplified (Fig. 4a3). This suggests that PD can promote the clearance of nuclear misfolded hAtx-1 in cultured cells and may explain the similar cytoplasmic redistribution we observed in fly brains (Fig. 3c). Interestingly, cytPC had the same effect on aggregation as the control DsRed, suggesting that PC does not facilitate the clearance of misfolded hAtx-1 protein even when it is present in the cytoplasm. These results indicate that PC and PD have different effects on nuclear polyQ aggregation: PC aggravates the nuclear aggregation of hAtx-1 with polyQ expansion, while PD can reduce the ectopically expressed toxic protein load.

Collectively, these experiments on polyQ aggregation in *Drosophila* and cell culture models show that *Dm*NMNAT isoforms generated from alternatively spliced mRNA variants have divergent neuroprotective properties. PD from RB variant has a robust neuroprotective ability, reduces hAtx-1[82Q] protein aggregation load and attenuates neuronal death, while PC from RA variant aggravates the nuclear aggregation of hAtx-1, increases neuronal apoptosis and exacerbates degeneration.

### PC and PD share biochemical properties with key differences

**Figure 5:**
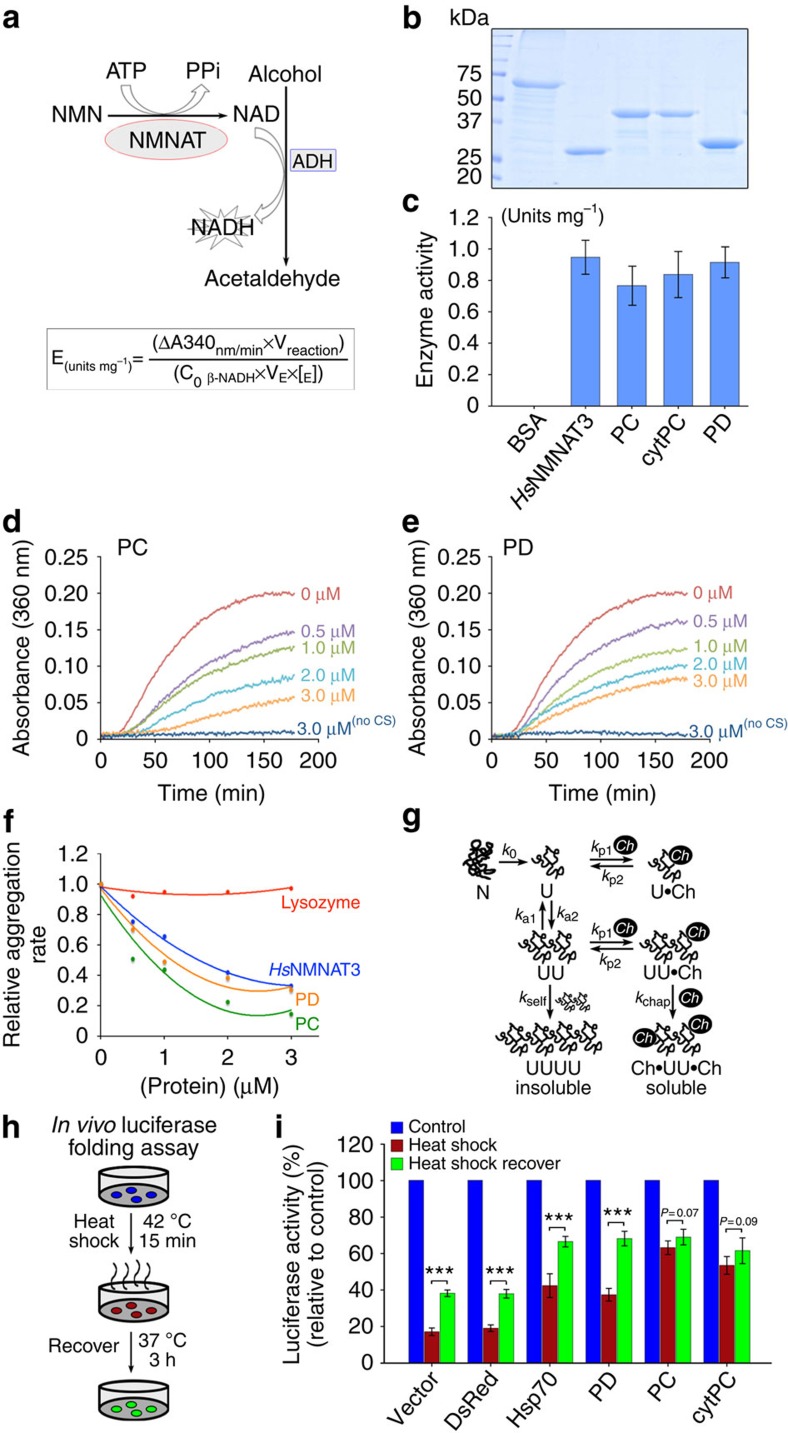
*Dm*NMNAT isoforms have similar but unique biochemical properties. (**a**) Diagram of the continuous coupled enzyme assay where NAD synthesized by NMNAT is reduced to NADH by alcohol dehydrogenase (ADH). NADH is monitored by absorbance at 340 nm. NMNAT activity is determined from the linear progression curve using the formula at the bottom. (**b**,**c**) NAD synthesis activity of recombinant *Hs*NMNAT3, PC, cytPC and PD (**b**) was measured by the continuous coupling assay as shown in **a**, with an overabundance of substrates in the reaction mix. Enzyme activity is calculated using the formula in **a** and presented as mean±s.d. *n*=4, triplicate sampling. (**d**–**f**) Aggregation of citrate synthase is prevented by addition of PC (**d**,**f**), PD (**e**,**f**) or *Hs*NMNAT3 (**f**) but not by lysozyme (**f**). Citrate synthase was denatured with heat at 43 °C, and the aggregation was monitored by absorbance at 360 nm. (**d**) The relative aggregation rate was calculated by the change in absorbance per minute and the aggregation rate of CS alone was set to 1. (**g**) A diagram of protein aggregation and its inhibition by chaperones. (**h**,**i**) HEK293T cells were co-transfected with luciferase and one of the protein expression plasmids as indicated. Forty-eight hours after transfection, HEK293T cells were subjected to heat shock as illustrated in the diagram (**h**). (**i**) Luciferase activity was measured without heat shock, after 15 min heat shock at 42 °C (red bars) or after 3 h recovery at 37 °C (green bars). Luciferase activity relative to no heat shock (set to 1) was displayed and presented as mean±s.d. ***P≤0.001 by Student *t*-test; *n*=4, triplicate sampling.

To dissect the mechanism underlying the divergent neuroprotective effects of *Dm*NMNAT isoforms, we next characterized the biochemical properties of PC and PD. Previous studies have shown that *Dm*NMNAT has NAD synthesis and chaperone activity^2^,^10^,^14^,^15^,^16^,^17^. We performed *in vitro* and in culture assays to determine these biochemical properties^14^. First, we isolated recombinant proteins of the *Hs*NMNAT3, *Dm*NMNAT-PC, -cytPC and -PD (Fig. 5b), and determined their NAD synthetic activity *in vitro* by a continuous coupled enzyme assay^48^ (Fig. 5a). We found that all NMNAT isoforms can synthesize NAD to a similar extent (Fig. 5c).

Next, we determined the chaperone function of PC and PD, both the ‘holdase'^49^ and ‘foldase'^50^ aspects of chaperone activity. First, we used an *in vitro* aggregation assay to directly measure the ability of recombinant PC and PD proteins to interact and prevent unfolded protein aggregation (Fig. 5g)^14^. As shown in Fig. 5d–f, both PC and PD prevented citrate synthase (CS) aggregation, with PC showing the lowest aggregation rate at all tested chaperone concentrations, suggesting that PC has holdase activity equal to or greater than that of PD. Second, we used the in-cell luciferase-refolding assay to measure the ability of *Dm*NMNAT isoforms to facilitate the refolding of unfolded luciferase after heat shock^14^ (Fig. 5h,i and Supplementary Fig. 4a,b). This assay allows the measurement of both luciferase unfolding during heat shock (red bars) and refolding after recovery (green bars). To minimize cell-type-specific effects, we performed the luciferase-refolding assay in mammalian HEK293T cells (Fig. 5) and *Drosophila* S2 cells (Supplementary Fig. 4) and obtained similar results. As shown in Fig. 5i and Supplementary Fig. 4b, PD expression protected luciferase from unfolding during heat shock and enhanced refolding significantly after heat shock, similar to that of positive controls mammalian heat shock protein 70 (Hsp70) and *Drosophila* Hsp83, the homologue of mammalian Hsp90 (ref. 51). Interestingly, PC greatly protected luciferase from unfolding during heat shock, but showed no significant refolding activity during recovery after heat shock compared with the other two groups. This difference was not caused by the localization of luciferase for two reasons: (1) luciferase was present in both the cytoplasmic and nuclear compartments, unaffected by the expression of Hsp83, PC or PD (Supplementary Fig. 4c), and therefore was accessible to both cytoplasmic PD (and Hsp70/Hsp83) and nuclear PC; (2) cytPC expression exhibited the same luciferase–holdase/foldase ability as PC, demonstrating a localization-independent property of PC.

The biochemical characterizations of the *Dm*NMNAT isoforms suggest that both spliced mRNA variants produce functional NAD synthases and chaperones. The protein isoforms share some aspects of biochemical properties but have distinct cellular characteristics. Specifically, RA produces nuclear protein PC with strong holdase activity, but with minimal refolding activity; RB produces cytoplasmic protein PD with strong refolding activity. Importantly, these specific cellular features of PC, that is, strong holdase but minimal refolding activity and nuclear localization, are consistent with its ability to enhance hAtx-1[82Q] aggregation when co-expressed. In contrast, PD showed refolding activity and is therefore capable of facilitating cellular clearance of unfolded proteins to reduce proteotoxicity.

### Differential regulation of *DmNmnat* variants under stress

The divergent neuroprotective outcome from overexpression of alternatively spliced isoforms posed the question of how the nervous system regulates the alternative splicing of *DmNmnat* endogenously. Previous work has found *DmNmnat* as a stress–response factor with its transcription regulated by heat shock factor (HSF)^35^. Both RA and RB originate from a common promoter region and share the first four exons; hence, their 5′ transcriptional regulatory elements are the same. However, we found that RA and RB are not equally expressed under normal conditions but at a ratio of 1.46:1 (RA/RB; Supplementary Fig. 5d), suggesting preferred expression of RA under normal conditions.

**Figure 6:**
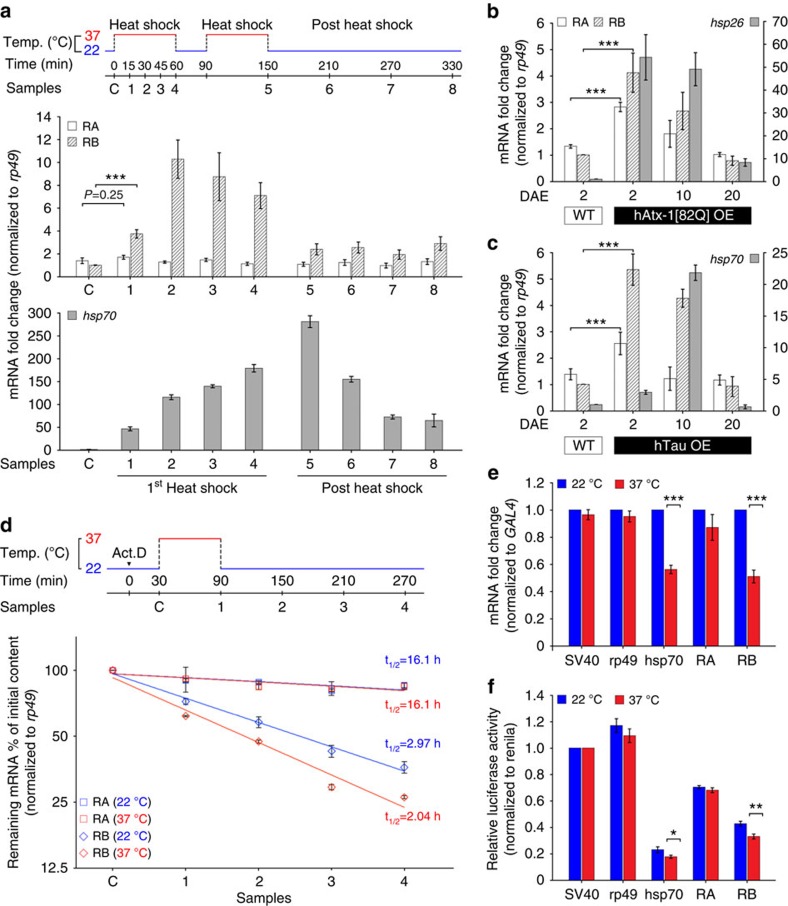
The mRNA variants of *DmNmnat* are differentially regulated under normal and stress conditions. (**a**) Wild-type flies (2 DAE) were subjected to heat shock and fly head samples were collected at indicated time points (diagram, top). Total RNA was extracted and the mRNA levels of RA, RB and *hsp70* were determined using qRT–PCR using variant-specific probes and normalized to *rp49* levels. The mRNA levels of RB and *hsp70* of the non-heat shock group (sample C) were set to 1, and mRNA fold change was displayed. (**b**,**c**) The mRNA levels of *DmNmnat* variants in wild-type flies, or flies overexpressing hAtx-1[82Q]- (**b**), or human Tau (hTau; **c**). Fold change relative to the RB, *hsp26* or *hsp70* mRNA levels of wild-type flies was displayed. (**d**) *Drosophila* S2 cells were pre-incubated with 10 μg ml^−1^ Act.D for 30 min, followed by 1 h of heat shock treatment. Samples were collected at indicated time points (diagram, top). qRT–PCR was performed to detect the remaining RA and RB mRNA levels at each time point and normalized to *rp49* levels. The mRNA level of sample C was set to 1. (**e**,**f**) *Drosophila* S2 cells were triply transfected with pAC-*GAL4*, pAC-*Renilla luciferase* and one of the following pUAST-*luciferase* reporter containing the indicated fragment from 3′ UTR of various genes: pUAST-*luciferase-SV40*, pUAST-*luciferase-rp49*, pUAST-*luciferase-hsp70*, pUAST-*luciferase-RA and* pUAST-*luciferase-RB*. At 72 h after transfection, the transcriptional inhibitor Act.D was added and cells were subjected to a 1-h heat shock. Then, cells were lysed and prepared for qPCR analysis (**e**) or luciferase activity (**f**). The mRNA level of *luciferase* was measured with qRT–PCR using a specific probe and normalized to the *GAL4* level (**e**). The mRNA level of *luciferase* under room temperature (22 °C, blue bars) is set to 1. (**f**) Luciferase activity was measured with the Dual-Glo Luciferase Assay System (Promega) using renilla luciferase as normalization control. The luciferase activity of the cells transfected with pUAS-*Luciferase-SV40* control vector is designated as 1. All data were presented as mean±s.e.m. *P≤0.05, **P≤0.01, ***P≤0.001, Student's *t*-test; *n*=4, triplicate sampling.

To examine how stress regulates the expression of *DmNmnat* variants, we determined the mRNA level of each variant under two stress conditions: heat shock stress (acute stress) and two proteotoxic stress (chronic stress) conditions induced by neuronal overexpression of either hAtx-1[82Q]^14^ or human Tau^16^. Interestingly, under all stress conditions tested, RA and RB showed distinct expression profiles. Under heat stress, RB was greatly increased immediately (within 15 min) on heat shock following a rise-and-fall pattern similar to that of Hsp70, while RA levels remained stable (Fig. 6a). Under proteotoxic stress, when either hAtx-1[82Q] or hTau was expressed in the nervous system, *DmNmnat* transcript expression, especially RB, was greatly increased compared with wild-type (*yw*) control (Fig. 6b,c). The rise in expression was similar to Hsp26 and Hsp70, suggesting that *DmNmnat* upregulation is a part of the endogenous stress response to proteotoxicity, consistent with previous observations^14^,^35^. Notably, in the late stage (20 DAE) of both proteotoxic stress models, both RB and heat shock protein (Hsp26 and Hsp70) transcripts were decreased (Fig. 6b,c). This is likely because of the sequestration of transcription factors by accumulated polyQ or Tau, similar to a previous report that found Hsp70 transcriptional upregulation in early stages of polyQ disease models and decline in expression in late stages^52^.

These results suggest that the expression of RA and RB are differentially regulated under normal and stress conditions. While RA is favourably expressed under normal conditions, RB is induced immediately on stress and its expression greatly surpasses that of RA. The rise-and-fall expression pattern of RB is similar to that of stress proteins such as Hsp70 and Hsp26. Such a differential expression of *DmNmnat* variants under stress can be achieved by regulating either 3′ untranslated region (3′UTR)-mediated mRNA stability or alternative splicing, or a combination of both.

To dissect the precise steps in RNA processing that are regulated by stress, we first determined the mRNA stability by measuring the decay rate of each variant under normal or stress conditions, using an actinomycin D (Act.D) chase assay^53^. As shown in Fig. 6d, the decay rate of RB under normal conditions (*t*_1/2_=2.97 h) was much faster than that of RA (*t*_1/2_=16.1 h). Under stress the degradation of RB increased significantly (*t*_1/2_=2.04 h), while RA remained stable (*t*_1/2_=16.1 h). These observations suggest that RA is more stable than RB, and stress further increases the decay rate of RB. Therefore, the differential expression of *DmNmnat* variants and the increased RB levels under stress conditions are unlikely because of a difference in RNA stability.

Since the 3′UTR sequences of RA and RB are quite distinct, we further determined the role of 3′UTRs in regulating the stability of *DmNmnat* variants under normal and stress conditions using a luciferase reporter assay in which the 3′UTRs of RA and RB were inserted into the 3′ end of the luciferase-coding sequence. The RA 3′UTR had minimal effect on the expression of luciferase mRNA under heat shock stress; however, the 3′UTR of RB resulted in a nearly 50% reduction in the luciferase mRNA expression level under stress, similar to that of the 3′UTR of *hsp70*, which has been reported to be unstable under heat shock^54^ (Fig. 6e). This effect is also reflected from the amount of functional luciferase protein translated from the mRNA, as the level of luciferase luminescence was significantly reduced under stress when the 3′UTR of RB was linked to luciferase (Fig. 6f). These results indicate that the 3′UTR is an important regulator of *DmNmnat* variants, and that stress decreases the mRNA stability of RB. However, differences in mRNA stability do not explain the observed levels of *DmNmnat* variants, specifically the increase in RB expression under stress conditions, despite the stress-induced instability because of its 3′UTR. The only possible step for the differential regulation of *DmNmnat* variants, then, is alternative splicing.

### Differential regulation of *DmNmnat* variants by splicing

**Figure 7:**
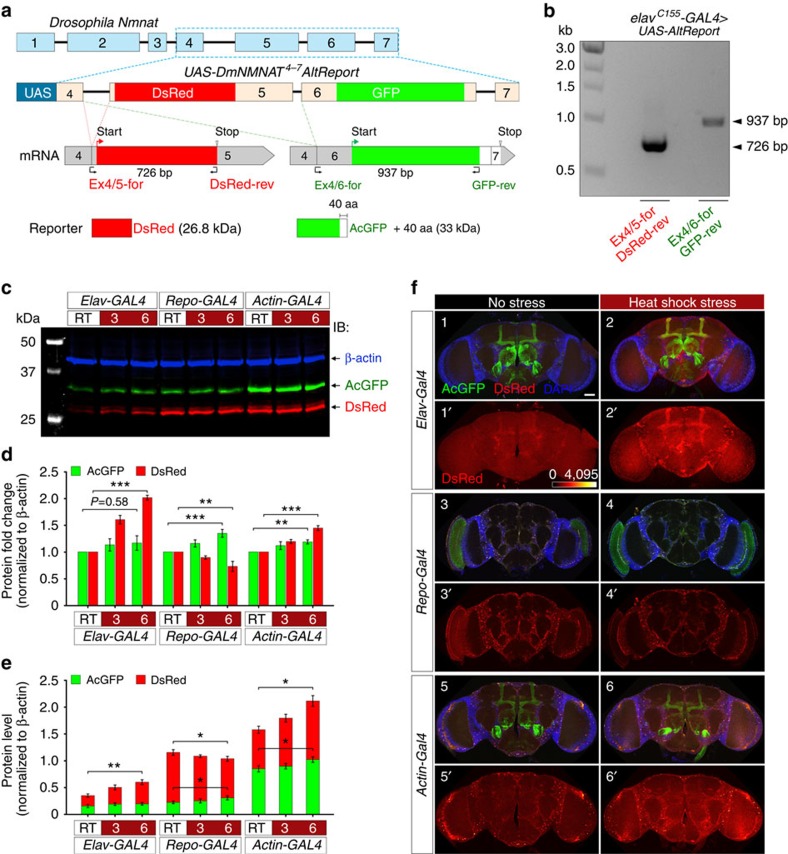
Alternative splicing of *DmNmnat* is regulated by stress in a cell-type-specific manner in the brain. (**a**) Construction of the alternative-splicing reporter line of *DmNmnat*. *UAS-DmNMNAT*^*4–7*^*AltReport* construct contains exons 4–7 with DsRed (red box) and AcGFP (green box) inserted in exons 5 and 6. The alternative-splicing reporter will generate two reporters DsRed (26.8 kDa) and AcGFP with a 40-amino-acid tail (33 kDa). (**b**) RT–PCR analysis of the total RNA extracts of 2 DAE fly heads from the alternative splicing reporter lines driven by *elav*^*C155*^-*GAL4* under normal conditions. The detection primers were indicated in **a**. (**c**–**e**) Western blot analysis (**c**) and quantification (**d**,**e**) of the protein extracts of 2 DAE fly heads from the alternative splicing reporter lines driven by *elav*-, *repo*- or *actin-GAL4* under normal (RT) or heat stress conditions at 3 and 6 h post-heat shock. Fold change relative to the level of RT groups is displayed. β-Actin was used as an internal control. Quantification of actual protein levels is shown in **e**. All data are presented as mean±s.e.m. *n*=4. *P≤0.05, **P≤0.01, ***P≤0.001 by one-way ANOVA *post hoc* Bonferroni test. (**f**) Adult flies (2 DAE) expressing the alternative splicing reporter by pan-neuronal driver *elav-GAL4* (1–2), pan-glial driver *repo-GAL4* (3–4) or ubiquitous driver *actin-GAL4* (5–6) were subjected to normal conditions or heat shock (1 h). Fly brains were dissected 6 h after treatment and imaged with confocal microscopy (AcGFP: green, DsRed: red, DAPI: blue). The expression level of DsRed is indicated separately with heat map (1′–6′). Scale bar, 50 μm.

To visualize and quantitatively analyse splicing events in the nervous system *in vivo*, we generated a reporter construct in pUAST vector spanning the alternatively spliced exons 4–7 of the *DmNmnat* genomic sequence (*UAS-DmNMNAT*^*4–7*^*AltReport*; Fig. 7a). This alternative splicing reporter also contains an AcGFP sequence inserted into exon 6 to serve as a proxy for splicing to RA, and a DsRed sequence inserted into exon 5 to indicate the splicing event that generates RB. After functionally confirming this reporter in cultured cells (Supplementary Fig. 6), we generated transgenic fly lines carrying *UAS-DmNMNAT*^*4–7*^*AltReport* and further confirmed its functionality *in vivo.* We designed primers bridging spliced exons (Fig. 7a) to amplify mRNAs produced through alternative splicing of the reporter using reverse transcriptase–PCR (RT–PCR). As shown in Fig. 7b, two transcripts were detected from brain extracts of flies expressing neuronal *DmNMNAT*^*4–7*^*AltReport* by *elav*^*C155*^-*GAL4*. These two fragments correspond to the predicted mRNA transcripts generated by unique splicing events. Therefore, *DmNMNAT*^*4–7*^*AltReport* is a functional tool for visualizing alternative splicing events *in vivo* in a tissue- and cell-specific manner and for determining quantitatively the ratio of RA versus RB being spliced.

To examine how stress affects splicing of *DmNmnat*, we first expressed the reporter in neurons by *elav-GAL4*, in glia by *repo-GAL4* or in all cells by *actin-GAL4*, and subjected the progeny reporter flies to heat shock stress (Fig. 7c). Because AcGFP and DsRed have similar protein stabilities with half-lives longer than the time of study (Clontech), the level of each reporter protein reflects the mRNA levels of RA and RB. Furthermore, any change of *elav*, *repo* and *actin* promoter activity in response to stress will affect both AcGFP and DsRed equally and will not confound the interpretation of splicing events detected by this reporter. Therefore, any change in the observed levels of AcGFP and DsRed can be attributed to a change in splicing. We first quantitatively determined the expression levels of AcGFP and DsRed under normal conditions (RT) and at 3 and 6 h post-heat shock (Fig. 7c–e). Interestingly, when the reporter was expressed in neurons, heat stress led to increased levels of DsRed (reporter for splicing to RB) by 100% without affecting the level of AcGFP (reporter for splicing to RA), suggesting that the splicing is shifted towards RB under stress. In glia, however, heat stress increased the level of AcGFP (RA) by 35% and concomitantly decreased the level of DsRed (RB) by 27%. When the reporter was expressed ubiquitously, we observed an overall slight increase in AcGFP (RA) and DsRed (RB) expression by 19% and 44%, respectively, in the whole brain (Fig. 7d,e). Next, we directly visualized the splicing reporters in the brain under normal and stress conditions. Consistent with the biochemical analysis, we observed a significant increase in DsRed (RB) signal in neurons, a reduction in glial cells, and a combined slight increase in the whole-brain post-heat shock (Fig. 7f).

Collectively, these data showed that first alternative splicing is the major step in RNA processing that results in the differential regulation of *DmNmnat* under stress in the brain; second, the effect of stress on *DmNmnat* pre-mRNA splicing differs in neurons and glia: under stress, neurons strongly prefer to splice to RB, while glia cells have a modest preference for RA. The latter also suggests that the remarkable upregulation of endogenous RB observed in the brain under stress (Fig. 6a–c) is contributed primarily by the neuronal population. This finding implies that the composition of *Dm*NMNAT isoforms is under cell-type-specific regulation and that PD isoform has distinct functional role in neurons, specifically under stress.

## Discussion

**Figure 8:**
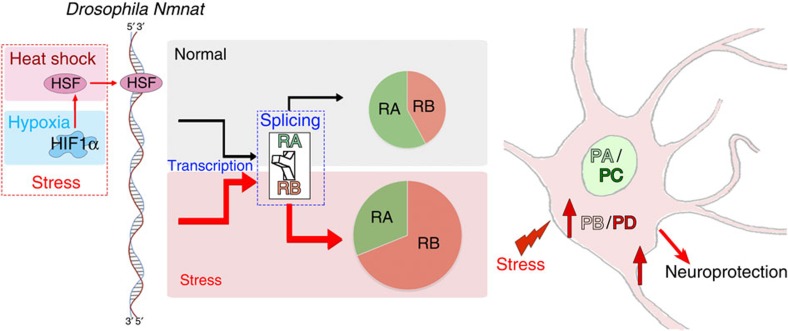
Schematic diagram of transcriptional regulation of *DmNmnat*. The transcription of *DmNmnat* is regulated by stress transcription factor HSF directly and HIF1α indirectly under heat and hypoxic stress. The splicing of pre-mRNA transcripts to RA and RB mRNA variants is regulated by stress in a cell-type-specific manner. In neurons, the splicing of pre-mRNA is shifted to variant RB under stress to promote the production of *Dm*NMNAT-PB/PD, therefore affording neurons with robust neuroprotective activity.

In this study, we identified a critical role of alternative splicing in regulating the maintenance capacity of the nervous system. Our results demonstrated that, through alternative splicing, two sets of protein products are generated from the *DmNmnat* gene. PC translated from RA is nuclear-localized, has strong ‘holdase' activity, minimum refolding activity and no neuroprotective activity against hAtx-1[82Q]-induced neurodegeneration. In contrast, PD translated from RB is localized to the cytoplasm, has strong refolding activity and robust protective activity against hAtx-1[82Q]-induced neurodegeneration. Since RA and RB are mutually exclusive, alternative splicing functions as a switch in *DmNmnat* expression between RA and RB: RA to produce nuclear protein isoforms, and RB to produce cytoplasmic neuroprotective factors (Fig. 8). Importantly, this switch is regulated by stress in the nervous system. Under normal conditions, both RA and RB are transcribed, with slightly more RA (RA:RB=1.46:1); therefore, both PC and PD proteins are expressed to sustain basic physiological needs. When neurons are under stress from either external (heat or hypoxia) or internal (proteotoxic) origin, the transcription of the *DmNmnat* gene is upregulated^35^ and the splicing of pre-mRNA is shifted to RB, therefore allowing the production of PD with robust neuroprotective activity (Fig. 8). Such regulation at the level of pre-mRNA splicing allows neurons to quickly respond to stress and achieve self-protection.

Stress response is a key process for all cells to maintain homeostasis. Central to the stress response is the increased synthesis of molecular chaperone (HSPs) that function to prevent protein misfolding and aggregation to maintain protein homeostasis^55^,^56^. On the onset of stress, the transcription of HSPs is quickly initiated by stress transcription factors such as HSFs or hypoxia-inducible factors^57^. The mRNA transcripts of inducible HSPs are often labile to facilitate rapid downregulation of the expression of HSP proteins after stress. Indeed, the half-life of Hsp70 or Hsp26 mRNA is less than 1 h (refs 58, 59), much shorter than those of some of the housekeeping genes; for instance, over 8 h for rp49 mRNA^60^. Previously, we have shown that *DmNmnat* is a stress–response factor and its transcription is upregulated under stress by the stress transcription factor HSF, similar to HSPs^35^. Interestingly, here we found that only RB is increased under stress, and *DmNmnat* variants have very different half-lives: RA has a long half-life of 16.1 h, and RB has short half-life of 2.97 h, which is further reduced to 2.04 h under stress (Fig. 6). Therefore, the transcription and regulation patterns of RA and RB fit the profiles of housekeeping genes and stress–response factors, respectively. Collectively, our findings present a model in which *DmNmnat* assumes two identities through alternative splicing: a housekeeping variant that is constitutively expressed with a long half-life and a stress–response variant that exhibits increased expression of unstable transcripts under stress. This mode of post-transcriptional regulation increases cellular tolerance to stress without adding new genes.

Mammals have three *Nmnat* genes, Nmnat1, -2 and -3, with distinct tissue expression and subcellular localizations^61^. It is possible that mammals achieve functional diversity of NMNAT through gene duplication rather than alternative splicing. For example, Human *NMNAT1* and -*2* are highly expressed in the nervous system, and NMNAT1 protein is nuclear-localized while NMNAT2 is cytoplasmic^61^. Both NMNAT1 and -2 have NAD synthetic activity and have been shown to be neuroprotective when expressed in the cytoplasm (see reviews^9^,^12^,^13^). Nuclear-localized NMNAT1 plays a role in preventing cell death after DNA damage, as its NAD synthetic activity is essential for the function of DNA repair enzymes such as PARP-1 (ref. 62). It is likely that the PC isoform is required in the nucleus for NAD metabolism, a hypothesis that requires experimental confirmation.

It is worth noting that the mechanism of transcriptional regulation by alternative splicing may be conserved between *Drosophila* and mammalian *Nmnat* homologues, as all three human *NMNAT* genes are multiexonic and predicted to be alternatively spliced. In a recent study, two splice variants of human *NMNAT3* (v1 and V3-FKSG76) have been experimentally identified^63^. It will be intriguing to investigate the alternative splicing and transcriptional regulation of human *NMNAT1* and -*2* in the nervous system. Exploring the role of alternative splicing in regulating neuronal maintenance and protection will prove both interesting and promising for the discovery of new therapeutic strategies for neuroprotection.

The regulation of alternative splicing occurs in multiple ways, including developmental stage-specific, sex-specific or tissue-specific manners and even in response to environmental^64^ or cellular (ER) stress^65^,^66^. Our findings also indicate that differential splicing events of *DmNmnat* could occur in a cell-type-specific manner. It has been reported that cell-specific alternative splicing not only participates in neuronal development^24^, but is also involved in human diseases such as cancer^10^ and neurodegeneration^7^. Recent studies have revealed that cell-specific alternative splicing in vertebrates involves repression in the inappropriate cell type as well as activation in the appropriate cell type^30^. Future work is required to identify the molecular components in the splicing machinery that sense and respond to stress in a cell-specific manner.

Maintaining neuronal homeostasis is a prerequisite for proper neurological activity. Healthy neurons are able to maintain their integrity throughout the life of an animal, suggesting the existence of a maintenance mechanism that allows neurons to sustain, mitigate or even repair internal or external damage. Our identification of alternative splicing functioning as a switch to enhance the neuroprotective role of *DmNmnat* indicates that the neuronal maintenance capacity is regulated endogenously and enhanced under stress for neurons to confer self-protection and higher resistance to adverse conditions.

## Methods

### Plasmid construction

Fifteen recombinant plasmids were generated for this study. They are pUAST-*luciferase-rp49*, pUAST-*luciferase-hsp70*, pUAST-*luciferase-RA*, pUAST-*luciferase-RB*, pUAST-*DmNMNAT*^*PA*^, pUAST-*DmNMNAT*^*PB*^, pUAST-*DmNMNAT*^*PC*^, pUAST-*DmNMNAT*^*cytPC*^ pUAST-*DmNMNAT*^*PD*^, pDsRed2-*PA*, pDsRed2-*PB*, pDsRed2-*PC*, pDsRed2-*cytPC*, pDsRed2-*PD* and *UAS*-*DmNMNAT*^*4–7*^*AltReport*. The primer sequences used in detection and cloning are listed in the Supplementary Table 2.

### Fly stocks and culture

Flies were maintained on a cornmeal–molasses–yeast medium and at room temperature (22 °C) with 60–65% humidity. The following *Drosophila* lines were obtained from the Bloomington Stock Center: *elav-GAL4*, *repo-GAL4*, *actin-GAL4* and *nervana-GAL4*. The *UAS-hAtx-1[2Q]*, *UAS-hAtx-1[30Q]* and *UAS-hAtx-1[82Q]* lines were obtained from Dr Huda Zoghbi's laboratory^14^.

### Stress paradigms

Adult female flies collected 24–48 h post eclosion were subjected to two 1-h heat shock at 37 °C with a 30-min room temperature period of acclimatization between shocks^35^. Following shock, the flies were collected at various time points. Both mRNA extraction and protein extraction were performed in preparation for further experiments.

### *Drosophila Nmnat* variant detection

Standard PCR was performed using the FailSafe PCR System (EpiCentre, Chichago, IL, USA) with the following amplification conditions: 25 cycles of 50 s at 95 °C, 50s at 60 °C and 1 min at 72 °C. The primers designed (Supplementary Table 2) for variant detection are common forward primer targeted the exon 1, and two distinct reverse primers targeted exon 5 and exon 7, respectively.

### RNA extraction and quantitative PCR

The samples for RNA extraction were collected from fresh or ultrarapid frozen fly heads or *Drosophila* S2 cells. Total RNA was extracted by TRIzol reagent (Invitrogen), according to the manufacturer's protocol. For each extraction, RNA concentration was measured spectrophotometrically at 260 nm, and 2 μg of RNA was used for reverse transcription reaction with a high-capacity cDNA reverse transcription kit (Applied Biosystems). Quantification of mRNA levels was performed using a CFX connect real-time detection system (Bio-Rad) and TaqMan probe-based gene expression analysis (Applied Biosystems). The amplification mix (20 μl) contained 100 ng of ssDNA reverse transcribed from total RNA, and 1 μl of gene-specific TaqMan probe–primer set. The samples were amplified by a PCR programme of 40 cycles of 10 s at 95 °C, 15 s at 55 °C and 1 min at 72 °C. The quantification of mRNA levels was carried out by the 2(-Delta Delta C(T)) Method^67^. A housekeeping gene, *rp49*, or *Gal4* was used as an internal control to standardize mRNA expression.

### Act.D chase assay

*Drosophila* S2 cells were incubated at room temperature for 30 min with 10 μg ml^−1^ of the transcriptional inhibitor Act.D (Amresco, Solon, OH, USA). After 30 m incubation with Act.D, Drosophila S2 cells were split equally into two groups. One was subjected to 1-h heat shock at 37 °C, and the other was retained at room temperature. At the indicated time points after the addition of Act.D, cells were harvested and total RNA was extracted. The expression levels of RA and RB at each time point were measured using qRT–PCR and normalized to the corresponding *rp49* levels. The remaining mRNA was determined by comparison with the expression level of the relevant gene at the zero time point when Act.D was added. Data were plotted on semi-log charts and exponential regression lines generated in Microsoft excel. The slope (*m*) of the regression lines was used to calculate the mRNA half-life (*t*_1/2_) using the equation *t*_1/2_=ln(2)/*m* to examine stability^53^.

### Luciferase reporter assay

*Drosophila* S2 cells were triply transfected in six-well plates using Cellfectin reagent (Invitrogen) with **pAC*****-GAL4***, **pAC-*****Renilla luciferase*** and one of the following pUAST-*luciferase* reporter containing the indicated fragment from 3′UTR of various genes: pUAST-*luciferase-SV40*, pUAST-*luciferase-rp49*, pUAST-*luciferase-hsp70*, pUAST-*luciferase-RA and* pUAST-*luciferase-RB*. At 72 h after transfection, the transcriptional inhibitor Act.D was added and cells were equally split onto two plates and one of the plates was subjected to a 1 h heat shock in an air incubator at 37 °C (which induced mild heat stress without unfolding luciferase). Then, cells were lysed and luciferase activity was measured with the Dual-Glo Luciferase Assay System (Promega) by means of luminescence measurements using a BMG plate reader.

### NMNAT recombinant protein expression

pET28b plasmids with NMNAT cDNA sequences were transformed into BL21DE3 bacteria and grown overnight. Expression of the recombinant protein was induced by the addition of isopropyl-b-D-thio-galactoside to the culture medium. The recombinant proteins were purified through His-select Ni-affinity gel (Sigma, St Louis, MO, USA) and Poly-Prep chromatography columns (Bio-Rad).

### Protein fractionation and western blot analysis

Proteins were extracted from fly heads with a homogenizing buffer (10 mM HEPES pH 7.9, 1.5 mM MgCl2, 10 mM KCl, 1 mM dithiothreitol (DTT) and 0.5 mM phenylmethylsulphonyl fluoride (PMSF)). Samples were spun at 14,000g for 30 min at 4 °C. Supernatant 1 was collected for later analysis. The pellets were resuspended with a protein extraction buffer (30 mM HEPES pH 7.9, 0.6 M NaCl, 1.5 mM MgCl2, 0.4 mM EDTA, 1 mM DTT, 25% glycerol, 1% NP-40 and 0.5 mM PMSF) and incubated at 4 °C for 30 min with vortexing every 6 min. Then the supernatant 2 was collected after 30 min centrifugation at 14,000g at 4 °C. Next, supernatants 1 and 2 were equally mixed and heated at 95 °C with 4 × Laemmli sample buffer. Lysates were probed with anti-DmNMNAT (1:8,000); Living Colors AcGFP Monoclonal Antibody (JL-8; 1:1,000, Clontech, Mountain View, CA, USA); Living Colors DsRed Polyclonal Antibody (1:1,000, Clontech); anti-cleaved caspase 3 (1:1,000, Cell Signaling, Beverly, MA, USA) and anti-β-Actin antibody (1:10,000, Sigma). Western blot analysis was carried out using the infrared dye-conjugated secondary antibodies, IR700 and IR800 (LI-COR Biosciences), and blots were imaged and processed on an Odyssey Infrared Imaging system.

### NMNAT enzymatic activity assay

The enzymatic activity of NMNAT was measured in a continuous coupled enzyme assay by monitoring the increase in absorbance at 340 nm caused by the reduction of NAD to NADH^48^. The reaction was performed at 37 °C in 16 nM semicarbazide-HCl, 0.625% (v/v) ethanol, 30 nM HEPES buffer (pH=7.4), 1.17 mM ATP, 15 U yeast alcohol dehydrogenase (Sigma), purified recombinant NMNAT protein variants and was initiated by adding nicotinamide mononucleotidase (NMN) to a final concentration of 0.625 nM. The activity is determined from the linear progression curve using the following formula:


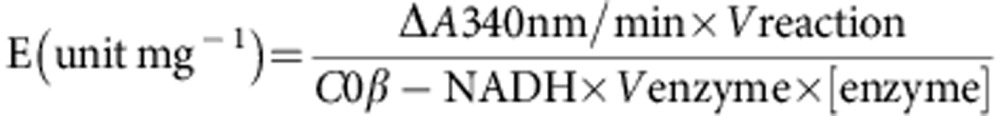


where *C*_*0*β-NADH_, the extinction coefficient of β-NADH at 340 nm, is 6.22.

### In-cell luciferase refolding assay

In mammalian cells, HEK293T cells were double-transfected in six-well plates using lipofectamine 2000 reagent (Invitrogen) with pCMV-*luficerase*, and one of the following plasmids: pDsRed2 vector, pCMV-*hsp70*, pDsRed2-*PC*, pDsRed2-cyt*PC* and pDsRed2-*PD*. At 48 h after transfection, the protein synthesis inhibitor cycloheximide was added and cells were subjected to a 15 min heat shock in an air incubator at 42 °C (which induced efficient unfolding of luciferase without killing the cells) and were then allowed to recover at 37 °C (ref. 68). In *Drosophila* cells, *Drosophila* S2 cells were triply transfected with pAC-*GAL4*, pUAST-*luciferase* and one of the following plasmids: pUAST vector, pUAST-*hsp83*, pUAST-*DmNMNAT*^*PA*^, pUAST-*DmNMNAT*^*PB*^, pUAST-*DmNMNAT*^*PC*^ and pUAST-*DmNMNAT*^*PD*^. At 72 h after transfection, the protein synthesis inhibitor cycloheximide was added and cells were subjected to a 15-min heat shock in an air incubator at 42 °C (which induced efficient unfolding of luciferase without killing the cells) and were then allowed to recover at room temperature^14^. Luciferase activity was measured with the Luciferase Assay System (Promega).

### *In vitro* CS aggregation assay

The substrate protein CS (Sigma) was mixed with egg white lysozyme (Sigma), or affinity-purified recombinant *Dm*NMNAT^PC^, *Dm*NMNAT^PD^ or *Hs*NMNAT3 proteins in HEPES (pH 7.4) buffer. Aggregation of denatured CS was induced by incubation at 43 °C. Aggregation was monitored by measuring the apparent absorption at 360 nm, owing to Rayleigh scattering of CS homoaggregates versus time. Measurements were performed on a FluoStar Optima plate reader (BMG Labtech). The relative chaperone activity of NMNAT was calculated as the scattering of CS aggregates with time versus NMNAT concentration^14^.

### In-cell aggregation assay

Cos-7 cells were co-transfected with lipofectamine 2000 (Life Technologies). After 48 h transfection, cells were fixed and stained with 4,6-diamidino-2-phenylindole (DAPI; blue) and visualized with an Olympus IX81 confocal microscope under × 60 magnification. DAPI Channel was thresholded to 400–4,095 and converted to binary signals to determine the size of nuclei. For the measurement of aggregates, the GFP channel was thresholded to 1,100–4,095. Aggregates are defined as GFP-positive objects of at least 0.4 μm^2^ and 3,000 intensity units.

### Immunocytochemistry of fly brains

Adult brains were fixed in PBS with 3.5% formaldehyde for 15 min and washed in PBS with 0.4% Triton X-100. Antibody dilutions used were as follows: anti-*Dm*NMNAT (1:500); anti-Ataxin-1 wild-type (1:500, NeuroMab, Davis, CA, USA); anti-Cleaved Caspase 3 (Asp175; 1:250, Cell Signaling); anti-nc82 (1:250, DSHB, East Iowa City, IA, USA) and secondary antibodies conjugated to Alexa 488, Alex 555 or Alex 647 (Jackson ImmunoResearch, Molecular Probes) were used at 1:250. DAPI (1:1,000, Invitrogen) staining was performed post-secondary antibody staining. All antibody incubations were performed at 4 °C overnight in the presence of 5% normal goat serum.

### Confocal image acquisition and processing

Confocal microscopy was performed with an Olympus IX81 confocal microscope and processed using FluoView 10-ASW (Olympus) and Adobe Photoshop CS6 (Adobe Systems, San Jose, CA, USA).

### Negative geotaxis behaviour assay

Ten age-matched female flies of the same genotype were placed in a vial marked with a line drawn horizontally 8 cm above the surface. The flies were gently tapped to the bottom surface and given 10 s to demonstrate climbing activity as a negative geotaxic response. After 10 s, the number of flies that successfully climbed above the 8-cm line was recorded. This assay was repeated 10 times and the averaged data were represented as percentages where the number of flies above the 8-cm mark was divided by the total number of flies tested within each group. For each negative geotaxis assay, a minimum of 10 groups (100 flies) per genotype was tested^69^.

## Additional information

**How to cite this article:** Ruan, K. *et al*. Alternative splicing of *Drosophila Nmnat* functions as a switch to enhance neuroprotection under stress. *Nat. Commun.* 6:10057 doi: 10.1038/ncomms10057 (2015).

## Supplementary Material

Supplementary Information Supplementary Figures 1-6 and Supplementary Tables 1-2
