# Supplementary material for: Alternative splicing of Drosophila Nmnat functions as a switch to enhance neuroprotection under stress
Source: Nat Commun. 2015 Nov 30;6:10057. doi: 10.1038/ncomms10057 (PMC4674693; doi:10.1038/ncomms10057)
Supplement: Supplementary Information — Supplementary Figures 1-6 and Supplementary Tables 1-2 [file ncomms10057-s1.pdf]

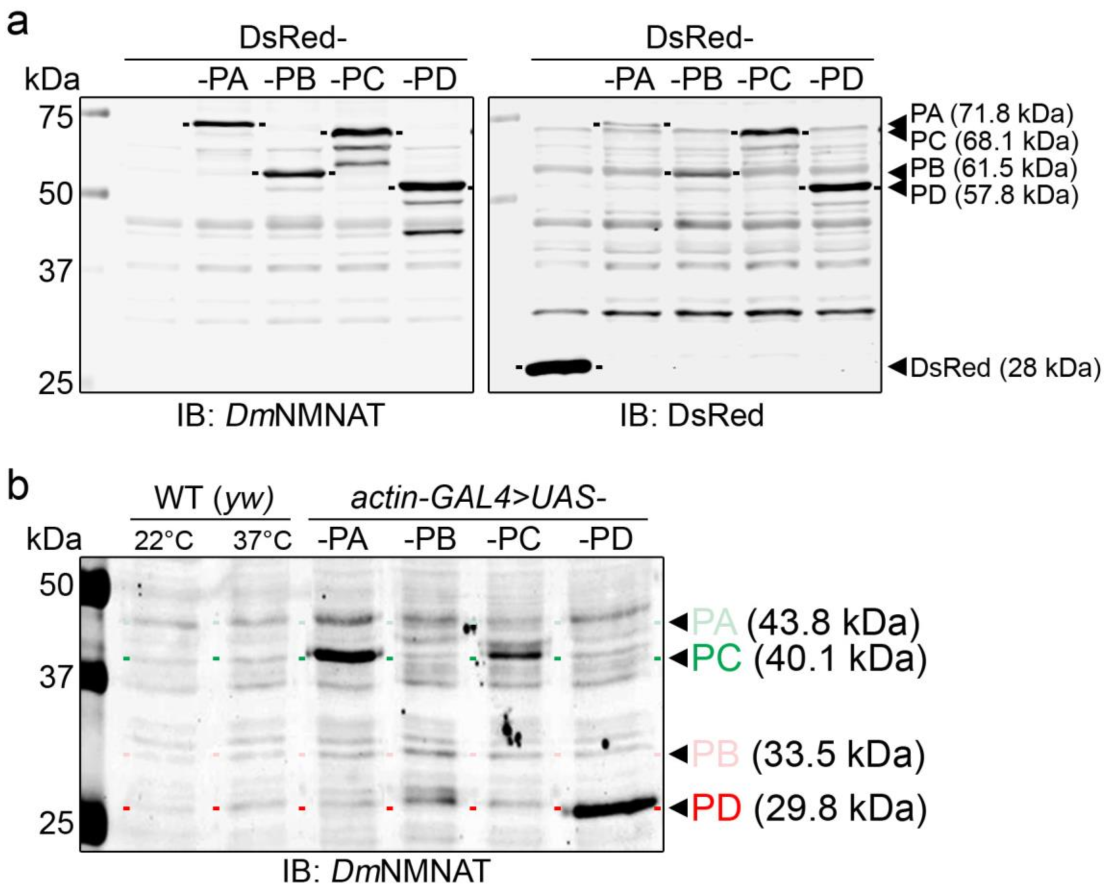

Supplementary Figure 1 *Drosophila* NMNAT polyclonal antibody specifically recognizes all NMNAT isoforms.

a, Western analysis using a polyclonal *Drosophila* NMNAT antibody[3] and a DsRed antibody of HEK293T cell lysates transfected with DsRed, DsRed-PA, DsRed-PB, DsRed-PC, and DsRed-PD constructs. **b**, Western analysis using *Drosophila* NMNAT antibody[3] of head extracts from wild-type flies (*yw*) under 22°C and 37°C, or flies overexpressing each of the four possible protein isoforms with ubiquitous *actin-GAL4* driver.

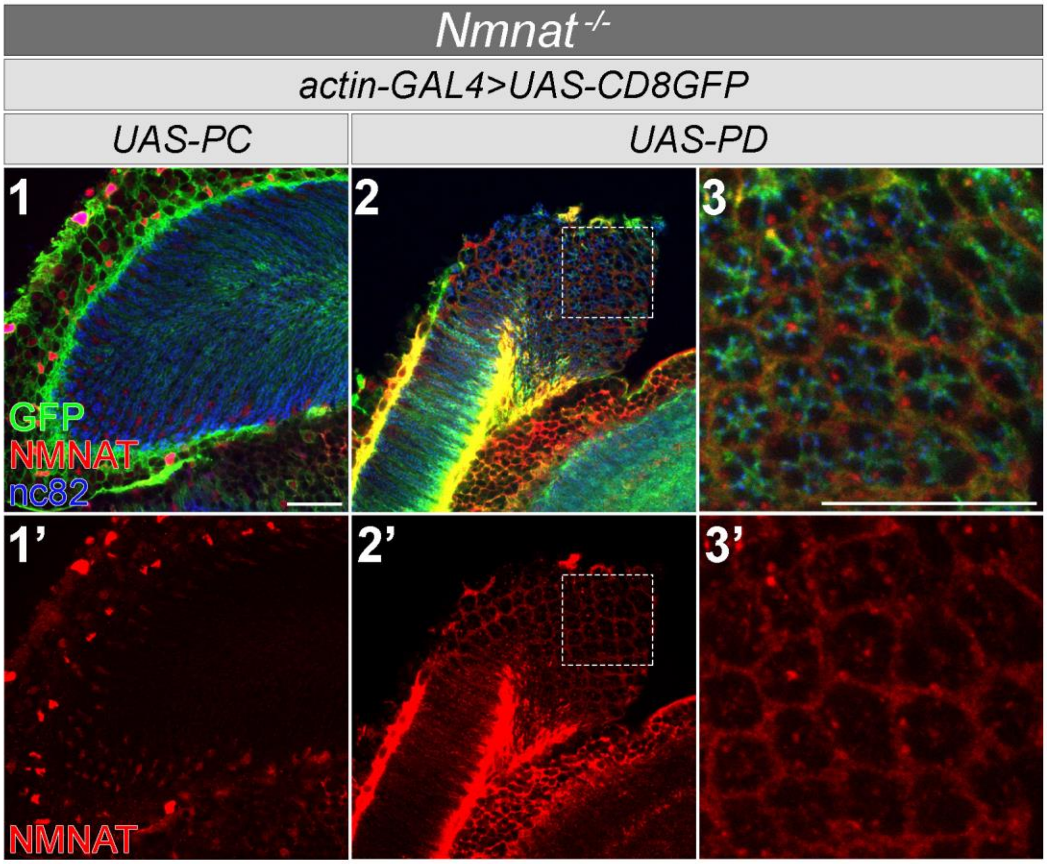

Supplementary Figure 2 *Dm*NMNAT protein isoforms are localized to distinct compartments in the lamina of the *Drosophila* visual system.

The adult *Drosophila* brain from *Nmnat*$^{-/-}$ null flies expressing CD8GFP (green) and PC (**1**) or PD (**2**, **3**) were stained for NMNAT (red), synapse marker nc82 (blue). The lamina structures were shown. Boxed areas in (**2, 2'**) are shown in (**3**, **3'**). Bottom row shows the NMNAT channel. Scale bars are 20 µm.

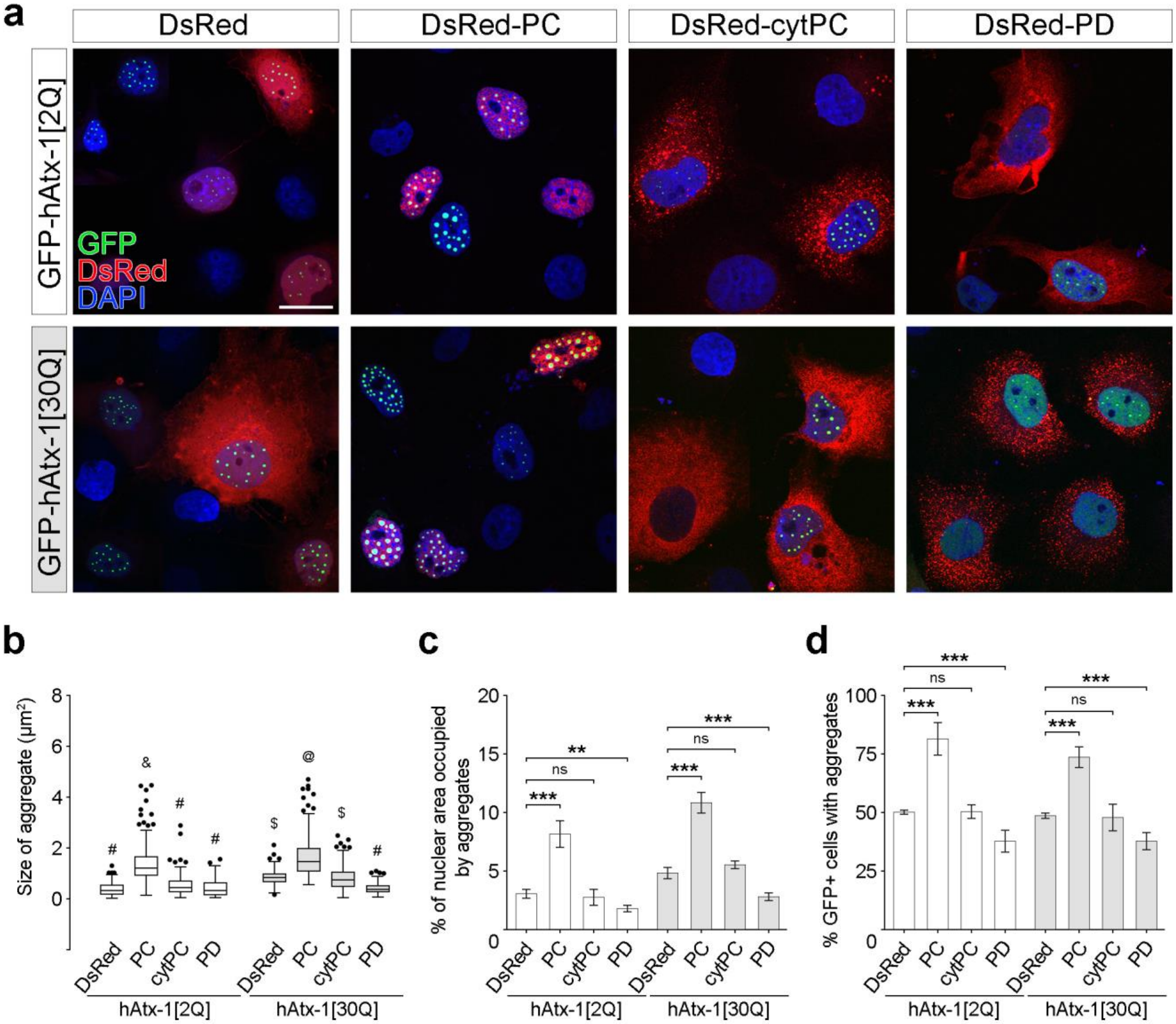
a
DsRed
DsRed-PC
DsRed-cytPC
DsRed-PD
GFP-hAtx-1[2Q]
GFP-hAtx-1[30Q]
GFP
DsRed
DAPI
b
Size of aggregate (μm²)
DsRed
PC
cytPC
PD
hAtx-1[2Q]
hAtx-1[30Q]
c
% of nuclear area occupied by aggregates
d
% GFP+ cells with aggregates
ns
***
**

Supplementary Figure 3 PC promotes the nuclear aggregation of hAtx-1.

a, PC promotes the nuclear aggregation of hAtx-1 with no polyQ expansion (hAtx-1[2Q]) or with sub-pathological (hAtx-1[30Q]). Cos-7 cells were co-transfected with GFP-hAtx-1[2Q] or GFP-hAtx-1[30Q] and DsRed, DsRed-PC, DsRed-cytPC, or DsRed-PD, and imaged at 48 h after transfection. DAPI (blue) stain was used to mark the nucleus. Scale bar is 20 µm for each row. **b-d,** Quantifications of aggregate size (**b**), percentage of nuclear area occupied by aggregates (**c**), and percentage of GFP-positive cells containing aggregates (**d**). (**b**) The size of aggregates was plotted with box and whisker plot. Box plots indicate the 25th percentile (bottom boundary), median (middle line), 75th percentile (top boundary), and nearest observations within 1.5-times of the interquartile range (whiskers). 200 aggregates were quantified for each condition. Different superscripts are statistically significant at $P \leq 0.05$, One-Way ANOVA post hoc Tukey's test. Nuclear area in (**c**) is defined by DAPI channel with a threshold of 400-4095. Aggregates are defined as objects at least 0.4 μm^2 and 3,000 intensity units. All data in (**c-d**) were presented as mean ± S.E.M., $n \geq 20$. Significance level was established by One-Way ANOVA post hoc Bonferroni test. ** $P \leq 0.01$, *** $P \leq 0.001$.

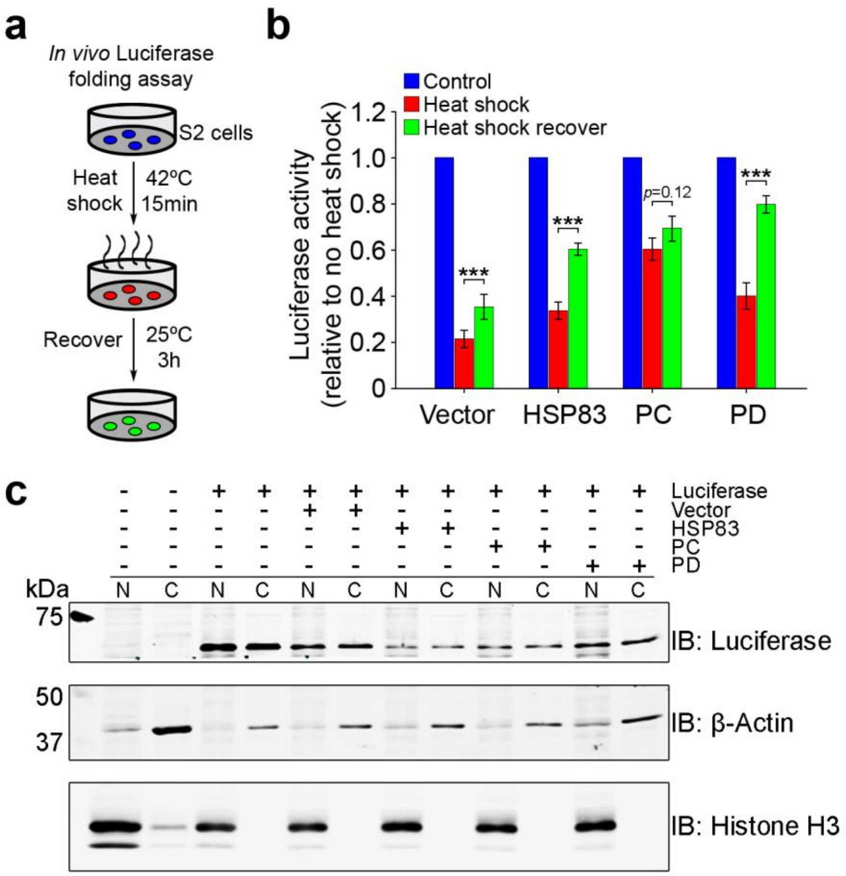

Supplementary Figure 4 PD has better refolding capacity compared to PC in luciferase refolding assay.

a, *Drosophila* S2 cells were co-transfected with luciferase and one of the protein expression plasmids as indicated. Seventy-two hours after transfection, S2 cells were subjected to heat shock as illustrated in the diagram. **b**, Luciferase activity was measured without heat shock, after 15 min heat shock at 42°C (red bars) or after 3 h recovery at 25°C (green bars). Luciferase activity relative to no heat shock (set to 1) was displayed and presented as mean ± S.E.M. *** $P \leq 0.001$ by Student t test; $n = 7$, triplicate sampling. **c,** Western analysis of luciferase protein from nuclear or cytoplasmic extractions of transfected or non-transfected *Drosophila* S2 cells.

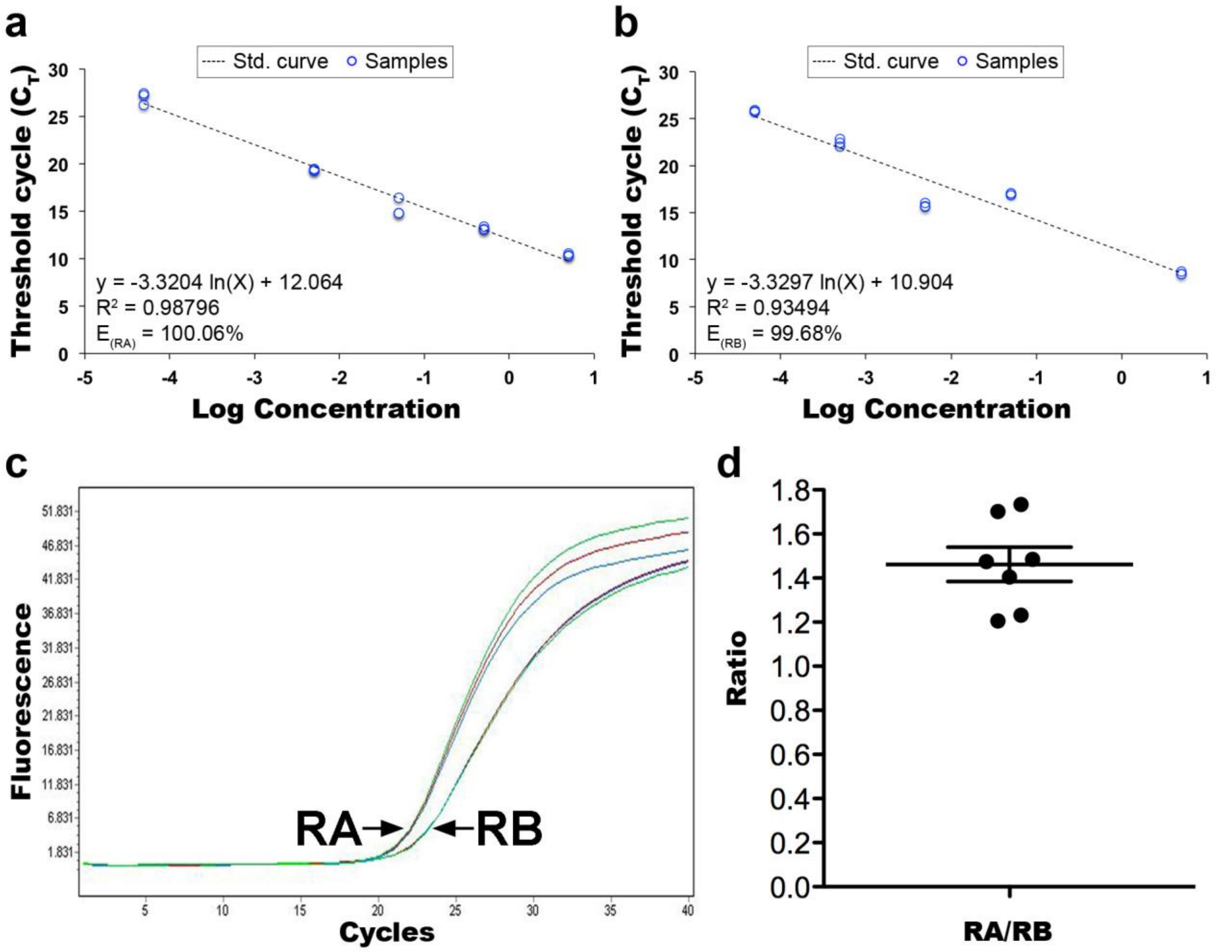

Supplementary Figure 5 The endogenous *DmNmnat* mRNA variant RA is expressed at a higher level than RB in the *Drosophila* brain under normal conditions.

a-b, Standard curves were generated using 10-fold dilution of cDNA templates amplified on the CFX connect real-time detection system (Bio-Rad). Standard curve with the C_T plotted against the log of the starting quantity of template for each dilution. The equation for the regression line and the r values are shown on the graph. The calculated amplification efficiencies were 100.06% (RA) and 99.68% (RB). **c**, The amplification profiles of the endogenous *DmNmnat* mRNA variants RA and RB under normal conditions. Quantitative real-time PCR using *DmNmnat* variant-specific probes was run on the total mRNA extract from the heads of wild-type adult female flies (2 DAE). The mean C_T values of RA and RB were 21.39 and 22.36 respectively. **d**, The scatter plot of the ratio between endogenous RA and RB in the brain under normal conditions. The ratio was calculated by the equation: $Ratio_{(RA/RB)} = 2^{C_T(RA) - C_T(RB)}$. The lines were presented as mean ± S.E.M., n = 7.

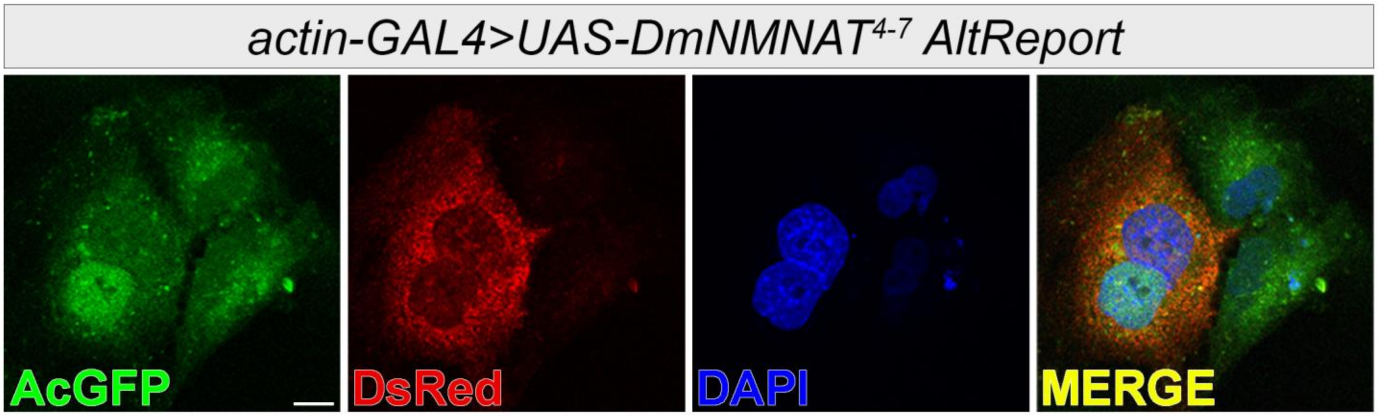

Supplementary Figure 6 The expression of alternative-spicing reporter (*DmNMNAT$^{4-7}$AltReport*) in cultured cells leads to distinct AcGFP or DsRed expression that indicates splicing events.

Cos-7 cells were co-transfected with *UAS-DmNMNAT$^{4-7}$AltReport* and pAC-*GAL4* plasmids, and imaged at 48 h after transfection. DAPI (blue) staining was used to mark the nucleus. Expression of AcGFP or DsRed indicates the inclusion of exon 6 or 5, respectively. Scale bar is 10 µm.

Supplementary Table 1 Single expression of *Dm*NMNAT cDNA variants suppresses the lethality caused by *Nmnat* loss-of-function.

**P**	☿	***yw;actin-GAL4/CyO;*** $\boldsymbol{Nmnat^{\Delta 4790\text{-}1}}$***/TM6,Tb***			
	♂	***yw;UAS-PA;*** $\boldsymbol{Nmnat^{\Delta 4790\text{-}1}}$ ***/TM3,Sb***	***yw;UAS-PB;*** $\boldsymbol{Nmnat^{\Delta 4790\text{-}1}}$ ***/TM3,Sb***	***yw;UAS-PC;*** $\boldsymbol{Nmnat^{\Delta 4790\text{-}1}}$ ***/TM3,Sb***	***yw;UAS-PD;*** $\boldsymbol{Nmnat^{\Delta 4790\text{-}1}}$ ***/TM6,Tb***
$\mathbf{F_1}$	**Expected Ratio**	**Rescued flies[1] : Total flies = 1:6 (16.7%)**			
	**Actual Ratio**	**10:72 (13.9%)**	**5:69 (7.2%)**	**5:52 (9.6%)**	**10:40 (25%)**
**%Rescue[2]**		**83.3%**	**43.5%**	**57.7%**	**>100%**

1. Genotype of rescued flies: *yw;actin-GAL4/DmNMNAT variants;* $Nmnat^{\Delta 4790\text{-}1}$/ $Nmnat^{\Delta 4790\text{-}1}$
2. %Rescue = Actual Ratio : Expected Ratio

Supplementary Table 2 A list of primers used in this study.

**mRNA detecting**	
**E1F**	5′-CATGCACCTTCGGATGTTCG-3′
**E5R**	5′-GCAAAGCGCGTCAAGAGTCG-3′
**E7R**	5′-GTCTTCCAGTTTCCTAAAGTTGC-3′
**3’UTR cloning**	
**F-RP49**	5′-CGTCTAGAGTGCTTAAGATTCTTGAGAGTTCTTG-3′
**R-RP49**	5′-CTGGATCCATCCAGATAATGCACATGTTATC-3′
**F-HSP70**	5′-CGTCTAGAGTGGCCAAAGAGTCTAATTTTTGTTC-3′
**R-HSP70**	5′-CTGGATCCAAGTCTACAAAACATTAAATGACAAG-3′
**F-RA**	5′-CGTCTAGAGTGAAACTGGAAGACGAATCTCAC-3′
**R-RA**	5′-CTGGATCCGGTAACGAACGTGTTTATTTTGC-3'
**F-RB**	5′-CGTCTAGAGTCGCGCTTTGCACGTGAAAAAG-3′
**R-RB**	5′-CTGGATCCAAGGTTATTGTTTAATGGGACTAC-3′
**cDNA cloning and mutagenesis**	
**F-PA/PB**	5′-GAATTCGAAAATGATTGTGAAAATCAGCTGGC-3′
**F-PC/PD**	5′-GAATTCGAAAATGTCAGCATTCATCGAGGAAA-3′
**R-PA/PC**	5′-GGTACCCTAAAGTTGCACTTGGGAAATC-3′
**R-PB/PD**	5′-GGTACCTCAAGAGTCGCATTCGGTCG-3′
**F-K349R**	5′-GAGCCAAGCAAGGAAGCAGAAGATTTCCCAAGTG-3′
**R-K349R**	5′-CTTCTGCTTCCTTGCTTGGCTCTCCTCTTTC-3′
**AltReporter cloning and detecting**	
**F-Ex4**	5′-CTCGAGAGCTCCACGTTGATTCGGCG-3′
**R-Ex7**	5′-GGTACCGGTAACGAACGTGTTTATTTTGC-3′
**Ex4/5-for**	5′-CTTTAAATCGCGGGATGCAC-3′
**Ex4/6-for**	5′-CTTTAAATCTAAGTATATAACGGA-3′
**DsRed-rev**	5′-AGATCTCTACAGGAACAGGTGGTGG-3′
**GFP-rev**	5′-GGATCCCTTGTACAGCTCATCCATGC-3′
